# NIR-Triggered Release of Nitric Oxide by Upconversion-Based Nanoplatforms to Enhance Osteogenic Differentiation of Mesenchymal Stem Cells for Osteoporosis Therapy

**DOI:** 10.34133/bmr.0058

**Published:** 2024-07-22

**Authors:** Xulu Ma, Zhao Luan, Qingxin Zhao, Anli Yang, Jinming Li

**Affiliations:** ^1^ MOE Key Laboratory of Laser Life Science & Institute of Laser Life Science, Guangdong Provincial Key Laboratory of Laser Life Science, Guangzhou Key Laboratory of Spectral Analysis and Functional Probes, College of Biophotonics, South China Normal University, Guangzhou 510631, China.; ^2^ Department of Breast Oncology, Sun Yat-sen University Cancer Center, State Key Laboratory of Oncology in South China, State Key Laboratory of Oncology in South China, Guangdong Provincial Clinical Research Center for Cancer, Guangzhou 510060, China.

## Abstract

Stem cell therapy is an attractive approach to bone tissue regeneration in osteoporosis (OP); however, poor cell engraftment and survival within injured tissues limits its success in clinical settings. Nitric oxide (NO) is an important signaling molecule involved in various physiological processes, with emerging evidence supporting its diverse roles in modulating stem cell behavior, including survival, migration, and osteogenic differentiation. To control and enhance osteogenic differentiation of mesenchymal stem cells (MSCs) for OP therapy, we designed a near-infrared (NIR) light-triggered NO-releasing nanoplatform based on upconversion nanoparticles (UCNPs) that converts 808-nm NIR light into visible light, stimulating NO release by light control. We demonstrate that the UCNP nanoplatforms can encapsulate a light-sensitive NO precursor, Roussin’s black salt (RBS), through the implementation of a surface mesoporous silica coating. Upon exposure to 808-nm irradiation, NO is triggered by the controlled upconversion of UCNP visible light at the desired time and location. This controlled release mechanism facilitates photoregulated differentiation of MSCs toward osteogenic lineage and avoids thermal effects and phototoxicity on cells, thus offering potential therapeutic applications for treating OP in vivo. Following the induction of osteogenic differentiation, the UCNP nanoplatforms exhibit the capability to serve as nanoprobes for the real-time detection of differentiation through enzymatic digestion and fluorescence recovery of UCNPs, enabling assessment of the therapeutic efficacy of OP treatment. Consequently, these UCNP-based nanoplatforms present a novel approach to control and enhance osteogenic differentiation of MSCs for OP therapy, simultaneously detecting osteogenic differentiation for evaluating treatment effectiveness.

## Introduction

Osteoporosis (OP) is a prevalent chronic systemic skeletal disease characterized by low bone mass, bone tissue degeneration, and disruption of bone microstructure [1]. With the aging of the population, the medical and socioeconomic impact of OP, particularly postmenopausal OP in those older than 50 years, is expected to increase [2]. The incidence of OP is steadily increasing worldwide, with an estimated 120 million Chinese individuals aged >50 years predicted to suffer from OP or osteopenia by 2050 [3]. Clinically administered medications to treat OP are classified into 2 categories: antiresorptive agents and bone formation-accelerating agents [4]. However, bisphosphonates, the most common anti-absorbent agents, can inhibit bone turnover, impair the natural mechanism of bone repair, and increase the risk of adverse complications such as jaw necrosis and atypical fractures. Furthermore, the efficacy of hormone replacement therapy decreases over time, and long-term treatment increases the risk of reproductive malignancies, cardiovascular diseases, and serious sequelae [5,6]. Therefore, developing a new treatment method for OP is crucial.

Mesenchymal stem cells (MSCs) are precursor cells commonly derived from bone marrow stroma and are capable of differentiating into chondrocytes, adipocytes, and osteoblasts [7,8]. Research has demonstrated that MSCs possess unique proliferation, differentiation, self-renewal, and survival abilities, rendering them an abundant and efficient source for the treatment of tissue and organ injury, inflammation, and other clinical conditions [9–11]. Moreover, previous studies have demonstrated that in vivo administration of MSCs has limited side effects. Various successful clinical approaches that involve the use of MSCs, including repairing craniotomy defects, bone grafts for orthopedic surgery, promoting healing after hip or knee replacement, and exogenous OP therapies, have been demonstrated [12,13]. Indeed, one such study by Ocarino et al. [14] involved intramural injection of differentiated rat bone marrow MSCs into the femur of osteoporotic female rats, which resulted in improved histomorphometric analysis and bone trabecular percentages compared with the untreated group. The MSC-based therapeutic approach has been a promising alternative for regenerative medicine, and the key point is, for example, how to effectively control and enhance osteogenic differentiation of MSCs for OP therapy.

Nitric oxide (NO) is a naturally occurring diatomic molecule synthesized by the body’s NO synthase, which plays various roles in physiological processes, including angiogenesis, wound healing, nerve transmission, bone reconstruction, and other biological activities [15,16]. NO has been shown to regulate the functions of actin, microfilament, and cells, inducing the differentiation of MSCs and the initiation of their further phenotypic differentiation [17,18]. Yang et al. [19] demonstrated that NO promotes stem cell osteogenic differentiation through signaling pathways, such as c-Jun N-terminal kinase (JNK)/mitogen-activated protein kinase (MAPK), and also balances osteoblast and adipocyte lineage differentiation through the JNK/MAPK signaling pathway in stem cells. Additionally, exogenous NO stimulates the proliferation of pre-osteoblasts [20]. However, the clinical use of NO is limited due to its short half-life (approximately 5 s) and limited diffusion radius (40 to 200 μm). Furthermore, NO cannot be released directly in cells [21–23]; thus, chemical strategies are necessary to improve NO storage and controlled release in vivo to regulate the differentiation of MSCs. Therefore, chemical strategies are needed to improve the storage and control of NO release in the body, with photocontrolled NO release considered an ideal approach. However, the use of ultraviolet (UV) excitation to release NO causes DNA base dimerization and affects normal DNA replication and synthesis, while the resulting phototoxicity causes irreversible damage to living cells; therefore, the use of visible light to produce NO is more suitable option [24].

Upconversion nanoparticles (UCNPs) are lanthanide-doped nanoplatforms with a unique trapezoidal electron energy structure; this structure allows control of energy transfer by selecting different lanthanide doping methods, converting low-energy incident NIR photons into high-energy visible light emission, and precisely adjusting emitted light to a desired wavelength with a maximum penetration depth [25]. UCNPs can be converted into UV and visible light from near-infrared (NIR) light, presenting advantages such as photostability, high chemical stability, low potential toxicity, large depth of light penetration, no background light interference, and minimal damage to biological tissues [26–28]. Due to their high specific surface area, high loading capacity, and good biocompatibility of the mesoporous structure, UCNPs coated with mesoporous silica have broad applications in biomedicine [29]. For instance, UCNP gas generation nanoplatforms based on the photocontrolled release of NO have been widely used in various fields, including cancer treatment, OP therapy, and antibacterial applications [30–32]. UCNPs have also been used to control differentiation of MSCs in vitro and in vivo by NIR light control [33,34].

**Fig. 1.**
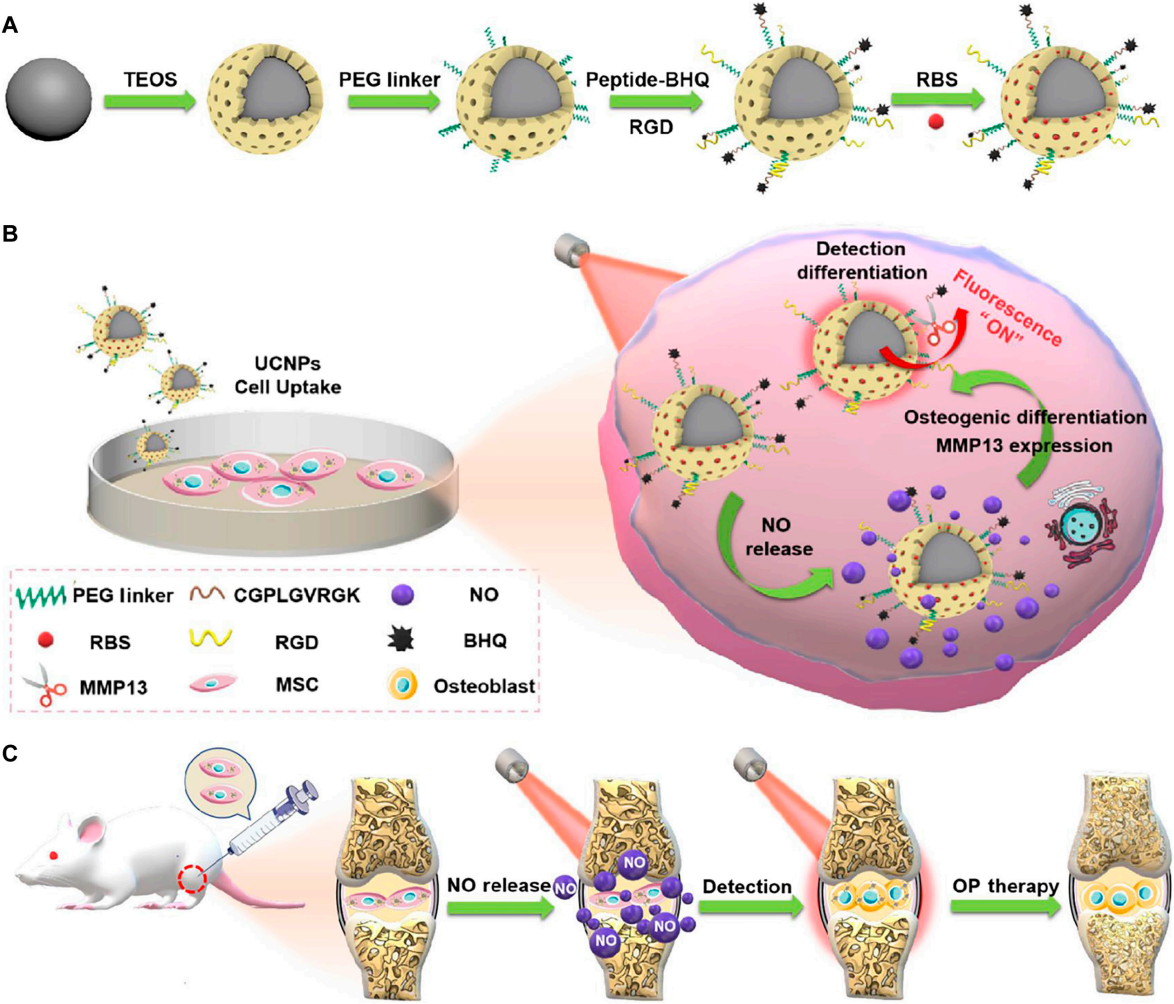
Schematic showing the method of synthesizing the multifunctional UCNP nanoplatform and its application in OP therapy. (A) Synthesis and structure of the multifunctional UCNP nanoplatform. (B) Operational principle of the multifunctional UCNP nanoplatform, which controls osteogenic differentiation through 808-nm NIR light-triggered release of NO and real-time detection of osteogenic differentiation in MSCs. (C) Injection of MSCs (containing UCNP nanoplatforms) into knee joint cavity of OP rat for OP therapy by NIR light.

In this study, we designed a NIR light-triggered NO-releasing nanoplatform based on UCNPs that converts 808-nm NIR light into visible light, stimulating NO release by NIR light to control and enhance osteogenic differentiation of MSCs for OP therapy, and simultaneously detect osteogenic differentiation as a means of evaluating the treatment effect on OP (Fig. 1). To construct the UCNP nanoplatform, Er^3+^/Nd^3+^-doped core–shell UCNPs (NaGdF_4_:Yb/Er@NaGdF_4_:Nd) were synthesized and coated with mesoporous silica (UCNP@mSiO_2_) to load a light-sensitive NO precursor, Roussin’s black salt (RBS). Next, the Arg-Gly-Asp (RGD) peptide (CGRGD) and the matrix metalloproteinase 13 (MMP13) detection group (CGPLGVRGK-BHQ_3_) were linked using NHS-PEG_4_-Mal to form the UCNP nanoplatforms (UCNP@mSiO_2_-peptide-BHQ). Upon loading of RBS, the UCNP/RBS nanocomplexes could be triggered by 808-nm NIR light irradiation and emit visible light to stimulate RBS and release NO, thereby controlling and enhancing osteogenic differentiation of MSCs in vitro and in vivo for OP therapy. Subsequently, when MSCs differentiated into osteoblasts, the newly generated MMP13 enzyme digested the detection peptide and removed black hole quencher (BHQ), leading to the recovery of the UCNP 650-nm fluorescence for detecting osteogenic differentiation to evaluate treatment effectiveness. Moreover, with an adjustment of the laser power, the release of NO could be precisely controlled, thereby promoting osteogenesis differentiation of MSCs in bone tissue and ultimately reversing OP.

## Materials and Methods

### Materials

Rare earth chlorides GdCl_3_·6H_2_O, YbCl_3_·6H_2_O, and ErCl_3_·6H_2_O were purchased from Sigma-Aldrich Reagent Ltd. (USA). Ethanol, cyclohexane, methanol, and other solvents were purchased from Guangzhou Chemical Reagent Factory (Guangzhou, China). *N*,*N*-Dimethylformamide (DMF; 99.8%), sodium hydroxide (NaOH; 97%), oleic acid (OA; tech grade, 90%), 1-octadecene (ODE; 90%), ammonium fluoride (NH_4_F; 98%), and tetraethyl orthosilicate (TEOS; 99%) were purchased from Aladdin Reagent Ltd. (Shanghai, China). The remaining chemicals were purchased from Aladdin Reagent Ltd. (Shanghai, China). All of the chemicals were of analytical grade. MAL-PEG_4_-NHS ester linker was purchased from Aladdin Reagent Ltd. (Shanghai, China). The CGRGD and CGPLGVRGK peptides were synthesized by Bank-peptide Biological Technology Co. Ltd. (Hefei, China). Phosphate-buffered saline (PBS), fetal bovine serum (FBS), Dulbecco’s modified Eagle’s medium (DMEM), and penicillin/streptomycin were procured from Gibco (Thermo Fisher Scientific, USA). Antibodies were obtained from Affinity Biosciences (USA). MSCs and osteogenic differentiation medium were purchased from Cyagen (Guangzhou, China). Alamar blue, 3-amino-4-aminomethyl-2′,7′-difluorescein, diacetate (DAF-FM) DA, 4′,6-diamidino-2-phenylindole (DAPI), BCIP/NBT Alkaline Phosphatase Color Development Kit, Alizarin Red S Staining Kit for Osteogenesis, and Nitric Oxide Assay Kit were purchased from Beyotime (Shanghai, China). Hematoxylin and eosin (H&E) and Masson’s trichrome stain kit were purchased from Solarbio (Beijing, China).

### Synthesis of core–shell UCNPs

The core of NaGdF_4_:Yb/Er was synthesized by a thermal decomposition method [35]. Briefly, a 50-ml 3-necked flask containing 15 ml of OA and 30 ml of ODE was added with 0.25 mmol of Yb(CH_3_CO_2_)_3_, 0.745 mmol of Gd(CH_3_CO_2_)_3_, and 0.005 mmol of Er(CH_3_CO_2_)_3_, heated at 160 °C to make a clear solution, and then cooled to room temperature. The solution was then treated with 10 ml of methanol containing 4 mmol of NH_4_F and 2.5 mmol of NaOH and stirred at room temperature for 0.5 h. The reaction vessel was then heated to 160 °C and degassed for 10 min to eliminate any remaining methanol and oxygen. It was then heated to 300 °C and kept under argon for 1 h. The reaction mixture was then cooled to room temperature, and the nanocrystals were precipitated by adding ethanol, centrifuged, washed 3 times with ethanol, and then dispersed in 5 ml of cyclohexane. At room temperature, 5 ml of cyclohexane containing 0.5 mmol of RE(CH_3_CO_2_)_3_ (RE = Gd/Yb/Nd = 60:10:20) was added to a 100-ml 3-necked flask holding 15 ml of OA and 30 ml of ODE and heated to 160 °C to make a clear solution. Core nanocrystals (5 ml) and methanol (10 ml) containing 4 mmol of NH_4_F and 2.5 mmol of NaOH were added when it cooled to room temperature. For 0.5 h, the yellow turbid solution was agitated at 50 °C. The mixture was then heated to 100 °C and degassed for 10 min to eliminate the methanol and oxygen. The solution was then heated to 300 °C and kept under argon for 1 h. After cooling the reaction mixture to room temperature, the core–shell UCNPs were precipitated by 3 separate ethanol additions, recovered by centrifugation, washed with ethanol, and then dispersed in 5 ml of cyclohexane.

### Synthesis of functionalized UCNPs

The cetyltrimethylammonium bromide (CTAB) (5 ml, 0.2 M) was added to the core–shell UCNPs (10 mg, dissolved in cyclohexane), agitated for 12 h, and then heated to 80 °C for 30 min to evaporate the cyclohexane before adding water (5 ml). After 2 h of alternating ultrasound and stirring, NaOH (150 μl, 0.1 M) was added and the mixture was vigorously swirled for 1 h. Ultrasonography and stirring were then repeated alternately for 2 h. A 20% volume portion (150 μl) TEOS ethanol solution was added, and the combination reacted for 1 day. (3-Aminopropyl)triethoxysilane (APTES) (2 μl) was added for 6 h in order to modify -NH_2_ for peptide coupling later on. UCNP@mSiO_2_-NH_2_ as produced was centrifuged, washed 3 times with ethanol and 1 wt % NaCl methanol solution, collected, and then dispersed in 5 ml of PBS. Then, the NHS-PEG_4_-MAL linker was conjugated on the surface of UCNP@mSiO_2_-NH_2_ by the reaction between -NH_2_ and -COOH. Briefly, 5 mg of NHS-PEG_4_-MAL was reacted for 12 h with 10 mg of UCNP@mSiO_2_-NH_2_. Centrifugation at 8,000 rpm for 5 min was used to extract the MAL-PEG functionalized UCNPs (UCNP@mSiO_2_-PEG-MAL), which were then washed 3 times with DMF. MMP13-sensitive peptide (CGPLGVRGK-BHQ, 2 mM in DMF, 200 μl), CGRGD peptide (2 mM in DMF, 200 μl), and diisopropylethylamine (DIPEA; 1 μl) were combined and stirred overnight to produce UCNP@mSiO_2_-peptide. After that, UCNP@mSiO_2_-peptide was allowed to react with BHQ_3_ (2 mM in DMF, 200 μl) and triethylamine (100 μl) for 24 h, followed by centrifugation at 8,000 rpm for 5 min and 3 ethanol washes to obtain UCNP@mSiO_2_-peptide-BHQ.

### Synthesis of RBS

RBS was synthesized ([NH_4_][Fe_4_S_3_(NO)_7_]) according to the previous work [36]. Briefly, NaNO_2_ (3.2 g) was dissolved in H_2_O (16 ml) and (NH_4_)_2_S (12.8 ml) was added. The solution was heated until it was dark brown after suddenly turning reddish brown. The aforementioned boiling solution (green) was brought to boiling after the addition of an 8-g aqueous solution of FeSO_4_·7H_2_O. To prevent NO leakage during this procedure, NH_4_OH (5 ml) was added. The solution was constantly heated for 15 min once it turned from black to brown, after it was promptly filtered. The solid product was vacuum freeze-dried and kept in the dark.

### Loading RBS to form UCNP/RBS nanocomplexes

RBS was loaded into UCNPs by an impregnation method. Typically, UCNP@mSiO_2_-peptide-BHQ (40 mg) and RBS (40 mg) were redispersed in 10-ml ethanol/water (1:1) solution under stirring and argon protection for 24 h in darkness. Then, the UCNP/RBS nanocomplexes (UCNP@mSiO_2_-peptide-BHQ/RBS) were collected by centrifugation at 8,000 rpm for 5 min and then washed with PBS 3 times. The solid product was freeze-dried under vacuum in a dark condition. The loading capacity was measured using UV-visible (UV-vis) absorption of RBS at 540 nm according to standard RBS curves at varying concentrations.

### Characterization

The UV-vis absorption spectroscopy was recorded on a UV-vis spectrometer (Lambda 35, Perkin-Elmer, USA) at room temperature. The upconversion fluorescence spectra were recorded by Edinburgh Instruments (FLS1000, UK). Fourier transform infrared (FT-IR) spectra were determined by an FT-IR spectrometer (Nicolet 6700, Thermo Fisher Scientific, USA) at room temperature. The transmission electron microscopy (TEM) images were collected on a high-resolution 2100F TEM (JEOL, Japan) operating at an acceleration voltage of 120 kV. The agarose gel electrophoresis system was from Junyi (Beijing, China). The Western blot system was a Bio-Rad Western blot system (Bio-Rad, Hercules, CA, USA). The gel imaging system was ChemiScope 6000 from Clinx (Shanghai, China) for Western blot results. The cell imaging was collected by NIB 900 inverted fluorescence microscope (Nexcope, Shanghai, China) and confocal microscope (Carl Zeiss LSM 880, Germany). Western blot results were determined by gel imaging system from Clinx (ChemiScope 6100, Clinx, Shanghai, China), and the animal imaging system was also from Clinx (IVScope 8000). The 808-nm NIR laser was from Yueyi Inc. (Guangzhou, China).

### NIR-triggered release NO detection

To detect the NIR-triggered release NO, the UCNP/RBS nanocomplexes (1 mg/ml) were seeded in 96-well plates. An aqueous solution of UCNP/RBS nanocomplexes was exposed to 808-nm NIR laser for a set period of time with varying power densities (0.5, 1.0, and 2.0 W/cm^2^). The NO released was measured using the commercial Griess reagent after laser irradiation. In brief, 1 mg of nanocomplexes was dissolved in 1 ml of PBS, and 50 μl of Griess reagent I solution was mixed with 50 μl of the aforesaid supernatant before being incubated in the dark following irradiation. Then, 50 μl of Griess reagent II was added for an absorbance measurement, and absorption value at 540 nm was recorded. The amount of NO emitted was measured using standard curve established by commercial NaNO_2_.

### UCNP nanoprobes detected the activity of MMP13 enzyme in buffer

UCNP@mSiO_2_-peptide-BHQ (500 μg/ml) was incubated at 37 °C for 30 min with various doses of MMP13 (0, 0.1, 0.02, and 0.04 mg). Using 0.04 mg of MMP13 at 37 °C for various times (10/20/30/40 min), the time-dependent experiment was also carried out. UCNP@mSiO_2_-peptide-BHQ was incubated at 0.04 mg (37 °C, 30 min) with a variety of proteins, including MMP3, MMP7, MMP13, and BSA, to confirm the specificity of the detection. The solution was examined after incubation using a fluorescence spectrometer and 808-nm laser (2 W/cm^2^).

### Cell culture and cell viability assay

The rat-derived MSCs were bought from Cyagen in Suzhou, China, and seeded in T25 cell culture flasks in DMEM containing 10% FBS and 1% penicillin-streptomycin at 37 °C in a humid environment with 5% CO_2_. To test the cytotoxicity of UCNP/RBS nanocomplexes, MSCs were routinely harvested by treatment with trypsin ethylenediaminetetraacetic acid solution (0.25%) and then seeded in 96-well plates. MSCs were subsequently treated with UCNP/RBS nanocomplexes for 24 h at various concentrations (0/10/20/50/100 μg/ml), and cytotoxicity was assessed using a microplate reader after addition of Alamar blue. Next, MSCs were incubated with UCNP/RBS nanocomplexes (100 μg/ml) at different incubation times (24/48/72 h) and then Alamar blue was added to test cytotoxicity with a microplate reader. Cell viability = sample (OD_570_)/control (OD_570_) × 100%.

### Intracellular NO staining

A commercial NO indicator called DAF-FM was used to stain NO in cells. At 10^4^ cells per well, MSCs were planted in 24-well plates. The DAF-FM solution (5 M) was applied to the cells at 37 °C for 20 min after the MSCs had attached themselves to them. The cells were subsequently exposed to 808-nm 1 W/cm^2^ radiation for 10 min after receiving UCNP/RBS nanocomplex treatment for 5 h. DAPI was used to stain the nucleus for 20 min at room temperature. The control group was UCNP therapy group. A laser scanning confocal fluorescence microscope (Carl Zeiss LSM 880, Germany) was used to observe cells.

### Western blot and ALP/ARS staining

The cells were seeded in 6-well cell culture plates and cultured for a certain time until at a density of about 2 × 10^6^ per well at 37 °C in 5% CO_2_ incubator. The cells were divided into 5 groups: control group (only cells), UCNP group (UCNP@mSiO_2_-peptide-BHQ 100 μg/ml), UCNP + RBS group (UCNP@mSiO_2_-peptide-BHQ 100 μg/ml, RBS 50 μg/ml), UCNP/RBS group (UCNP@mSiO_2_-peptide-BHQ/RBS 100 μg/ml), and UCNP/RBS + NIR group (UCNP@mSiO_2_-peptide-BHQ/RBS 100 μg/ml, and 20 min, 1 W/cm^2^ NIR irradiation). The cells were grown continuously for 7 days with osteogenic differentiation medium to establish differentiation for Western blot after being incubated with supplies for 4 h and rinsed with PBS. MSCs were then washed and lysed with cold cell lysis buffer and immunoprecipitation lysis buffer. The cell lysates were incubated on ice for 30 min before being centrifuged at 10,000 rpm for 20 min. The protein content was determined using the BCA Kit. An equivalent amount of protein was separated using sodium dodecyl sulfate–polyacrylamide gel electrophoresis (SDS-PAGE) and put to a polyvinylidene difluoride (PVDF) membrane. After incubating with 5% skim milk containing a 1:2,000 dilution of the primary antibody, the membrane was shaken horizontally at 4 °C overnight before being treated with a secondary antibody conjugated to horseradish peroxidase (HRP). Following treatment with an improved chemiluminescence detection kit, the bands were identified using ChemiScope 6000 and their intensity was measured using ImageJ. The alkaline phosphatase (ALP)/alizarin red S (ARS) staining was performed to observe osteogenic differentiation following treatment with various groups. The MSCs were statically seeded at a density of 2 × 10^4^ cells per well on 24-well culture plates and incubated for 10 days at 37 °C in 5% CO_2_. Following that, the growth medium was withdrawn and the MSCs were rinsed with sterile PBS and fixed with 4% paraformaldehyde for 30 min individually. Next, the fixed MSCs were washed with PBS and then incubated with ALP or ARS staining buffer 30 min for ALP/ARS staining. Finally, the cells were washed with PBS and photographed with a microscope.

### Real-time detection of osteogenic differentiation in MSCs by UCNP nanoprobes

In 24-well cell culture plates, the cells were plated and cultured for a certain time until they reached a density of 10^5^ cells per well. Five sets of cells were created and treated as part of a Western blot experiment. The cells were grown for 1, 3, and 7 days, respectively, after being treated with supplies for 4 h. The cells were then fixed with 4% paraformaldehyde for 30 min after being rinsed 3 times with PBS. The MSCs were washed 3 times with PBS before being stained with DAPI and given a 30-min incubation with Actin-Tracker Phalloidin. NIR inverted fluorescence microscopy was used to capture the green (540 nm) and red (650 nm) fluorescence from UCNPs.

### OP molding

Eight-week-old female Sprague-Dawley rats weighing an average of 200 ± 10 g were purchased from Guangdong Medical Laboratory Animal Center (Guangzhou, China). All animal experiments were approved by the Institutional Animal Care. High dosages of dexamethasone, which have been known to be able to generate OP, were injected into the right thigh muscle of Sprague-Dawley rats for OP modeling [37]. Rats were split into 2 groups: the Sham group and the OP group. Dexamethasone was administered to OP rats at a dose of 1 mg/kg, while phosphate-buffered saline (PBS) treatment was given to normal rats. For 4 weeks, intramuscular injections were given to both rats in both groups twice a week.

### Micro-CT scanning

The OP rats were randomized and divided into 4 groups (*n* = 4): OP (only inject MSCs), UCNP + RBS (MSCs were incubated with 100 μg/ml UCNP@mSiO_2_-peptide-BHQ and 50 μg/ml RBS), UCNP/RBS (MSCs were incubated with 100 μg/ml UCNP@mSiO_2_-peptide-BHQ/RBS), and UCNP/RBS + NIR (MSCs were incubated with 100 μg/ml UCNP@mSiO_2_-peptide-BHQ/RBS and 20-min NIR irradiation with 1 W/cm^2^). The treated MSCs were injected 0.2 ml twice weekly for a total of 2 weeks, with illumination beginning 1 day following injection, into the right joint cavity of OP rats. The sham group received merely physiological saline injections. The rats were euthanized after 8 weeks of therapy, and the femur was removed for micro-computed tomography (CT) bone examination. Three rats were randomly chosen from each group after the rats were sacrificed, and the femur’s bone tissues were taken out and treated in a 4% paraformaldehyde solution. Micro-CT (SkyScan1276, Bruker, Germany) was used to scan the left femur metaphysis and the third vertebral body’s trabecular microarchitecture. NRecon software was used to reconstruct the images. The 3-dimensional (3D) images and the bone morphometric parameters of bone mineral density (BMD), bone surface/tissue volume (BS/TV), bone volume fraction (BV/TV), trabecular separation (Tb.Sp), and trabecular number (Tb.N) were determined by analyzing the volume of interest (VOI). Then, using the Mimics program, BMD, BV/TV, Tb.N, Tb.Sp, trabecular thickness (Tb.Th), Tb.Sp, and trabecular pattern factor (Tb.pf) were measured.

### Histopathological, immunohistochemical, and immunofluorescence assays

The femur was cut (4 μm in thickness) after micro-CT and then subjected to histological, immunohistochemical, and immunofluorescence staining. H&E and Masson’s trichrome staining were used to determine the new bone development in the femur in accordance with the manufacturer’s instructions, and then stained sections were examined with a camera (DS-Ri1-U3, Nikon, Japan). To gauge the degree of osteogenesis, immunohistochemical labeling was done for the expressions of Runt-related transcription factor 2 (Runx2), bone morphogenetic protein 2 (BMP-2), and osteopontin (OPN). First, 3% hydrogen peroxide was applied to the sections to inhibit endogenous peroxidase, and then 3% bovine serum albumin (BSA) was used to prevent nonspecific binding. Proteins were identified using a primary antibody in diluted concentrations of 1:500. After being dehydrated in ethanol/xylene and fixed with permanent medium, antibody complexes were detected with the Polink-1 HRP 3,3′-diaminobenzidine (DAB) detection system and photographed using a confocal microscope. OPN and BMP-2 immunofluorescence were carried out to determine the degree of osteogenesis. To avoid nonspecific binding, the sections were first treated with EDTA antigenic repair solution (pH 8) to repair antigen and 3% albumin from bovine serum (BSA). After incubating at 4 °C overnight with PBS containing a 1:1,000 dilution of the primary antibody (Abcam, USA), fluorescent secondary antibodies of the same genus as the primary antibody were added. The nucleus was then stained with DAPI and imaged using a confocal microscope.

### Real-time detection of osteogenic differentiation of MSCs in OP rats

The cells were sown on 24-well cell culture plates and grown for a period of time until they reached a density of 100,000 cells per well. Then, the cells were split into 4 groups: UCNP (UCNP@mSiO_2_-peptide-BHQ 100 μg/ml), UCNP + RBS (UCNP@mSiO_2_-peptide-BHQ 100 μg/ml, RBS of 50 μg/ml), UCNP/RBS (UCNP@mSiO_2_-peptide-BHQ/RBS of 100 μg/ml), and UCNP/RBS + NIR (UCNP@mSiO_2_-peptide-BHQ/RBS 100 μg/ml, and 20-min NIR irradiation with 1 W/cm^2^). Following that, MSCs were harvested and treated with different groups before being injected into the right knee joint of rats (100 μl) and then irradiated. The fluorescence imaging of rats at the right knee joint in UCNP, UCNP + RBS, UCNP/RBS, and UCNP/RBS + NIR groups was recorded after 1, 3, and 7 days using an animal imaging system (chemiscope8800, Clinx, Shanghai, China) with an excitation 808-nm laser (1 W/cm^2^) at 540/650-nm fluorescence emission.

### Statistical analysis

Experimental data were repeated at least 3 times and expressed as means ± SD. Statistical marked was evaluated using Student’s *t* test. The value of *P* < 0.05 (*), *P* < 0.01 (**), and *P* < 0.001 (***) was considered statistically significant.

## Results

### Synthesis and characterization of multifunctional UCNP nanoplatforms

The 808-nm NIR-responsive UCNPs with a core–shell structure were synthesized using a thermal decomposition method. Initially, the UCNP core (NaGdF_4_:Yb/Er) was synthesized, before enhancing the upconversion luminescence (UCL) performance by enabling the excitation of 808-nm NIR light and coating the shell layer containing Nd. The UCNPs exhibited a monodispersed hexagonal shape and a uniform size of approximately 50 nm of TEM image (Fig. S1A) with strong UCL (Fig. S1B). Next, a layer of mesoporous SiO_2_ was coated on the surface of UCNPs (UCNP@mSiO_2_) to make them hydrophilic and use them as nanoplatforms for loading the NO donor RBS. The composition of UCNPs and UCNPs@mSiO_2_ was analyzed using x-ray diffraction (XRD), and the patterns are shown in Fig. S2. The XRD analysis revealed that all of the diffraction peaks ascribed to the pure hexagonal phase known from β-NaGdF_4_ (JCPDS No. 027-0699), and the broad peak at 2θ = 32° of amorphous mSiO_2_ could be observed.

**Fig. 2.**
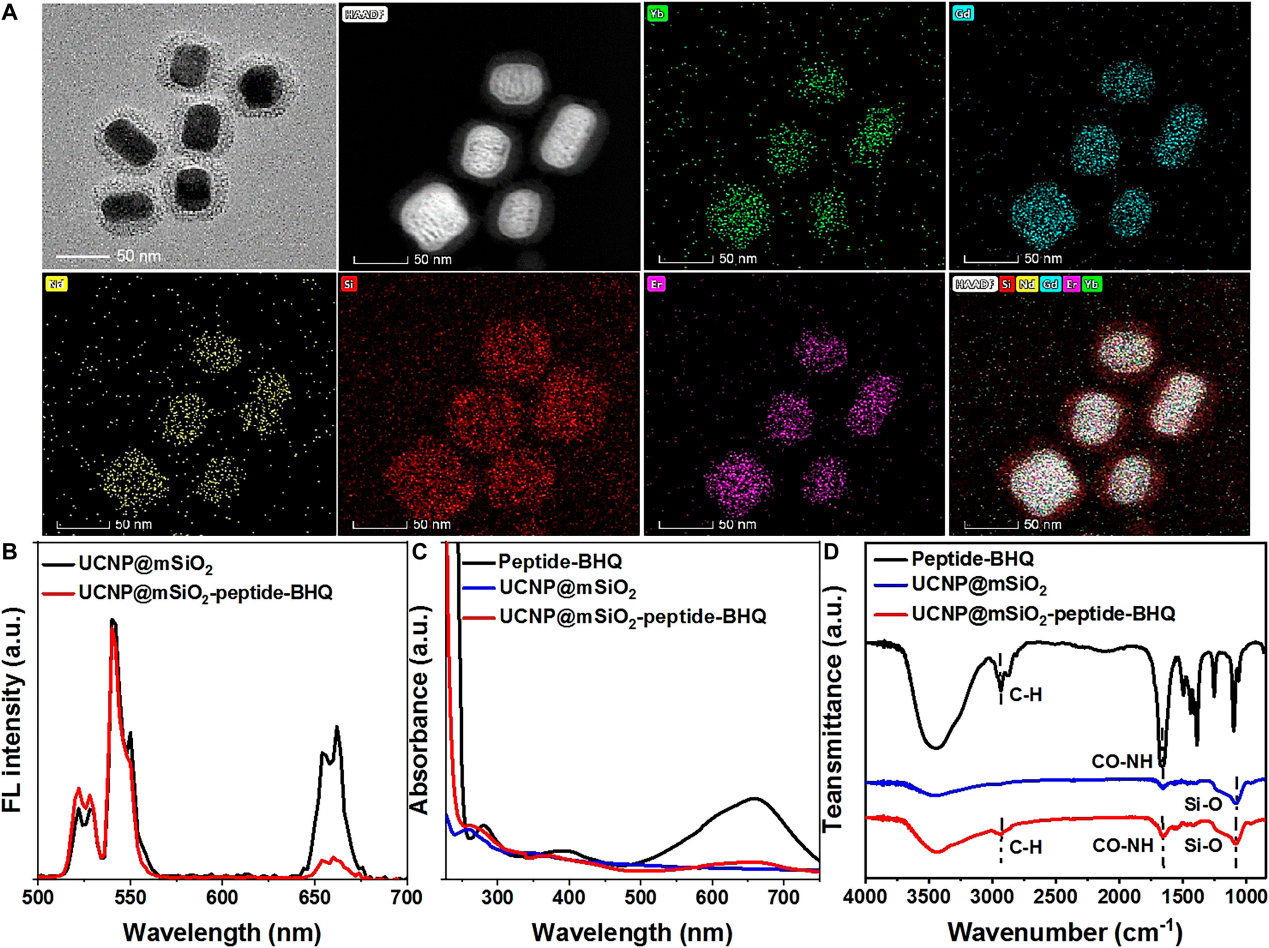
Characterization of the UCNP nanoplatforms (UCNP@mSiO_2_-peptide-BHQ). (A) TEM image and corresponding elemental mapping images of UCNP nanoplatforms. (B) UCL spectra of the UCNP@mSiO_2_ and UCNP nanoplatforms. (C) UV-vis absorption spectra of the peptide-BHQ, UCNP@SiO_2_, and UCNP nanoplatforms. (D) FT-IR spectra of the peptide-BHQ, UCNP@SiO_2_, and UCNP nanoplatform.

Next, the RGD peptide (CGRGD) and the MMP13 enzyme detection group (CGPLGVRGK-BHQ_3_) were linked using NHS-PEG_4_-Mal to create multifunctional UCNP nanoplatforms (UCNP@mSiO_2_-peptide-BHQ). TEM was used to observe the porous morphology of various regions of the encapsulated mSiO_2_. The TEM image revealed the mean pore sizes in the outer silica coating and interior UCNP structure regions to be 9.0 ± 2.3 and 60 ± 6.2 nm, respectively (Fig. 2A). The distinct hexagonal 3D structure of UCNPs is reflected in morphological alterations that have the potential to be reverted during later modification operations. Element mapping was used for homogeneous distribution analysis of the Yb, Gd, Nd, and Er elements, confirming the composition of the core–shell UCNPs and the presence of the mSiO_2_ coating (Fig. 2A). The UCL spectra of the UCNP@mSiO_2_ and UCNP nanoplatforms were measured, as shown in Fig. 2B. The UCL intensity markedly increased after coating the Nd^3+^ shell, which facilitated effective excitation of RBS and allowed simultaneous detection of MMP13 enzyme activity through 808-nm NIR irradiation. Figure S3 showed the UV absorption peak of BHQ_3_ and its structural formula. As a result, the UCL signal of the UCNPs nanoplatform is practically unaffected at 540 nm but is quenched at 650 nm by BHQ_3_, which was attributed to the BHQ quenching effect on absorption at 600 to 700 nm. UV-vis further confirmed the formation of the UCNP nanoplatforms (Fig. 2C), showing strong UV adsorption from 500 to 700 nm near BHQ for the MMP13 detection group (peptide-BHQ). Moreover, FT-IR detected functional groups (Fig. 2D), with peptide-BHQ exhibiting peaks at 2,923/2,849 cm^−1^ (benzene ring -CH_2_- stretching vibration) and 1,553 cm^−1^ (CO-NH amide bond). UCNP@mSiO_2_ revealed a silicon–oxygen bond group at 1,095 cm^−1^, while the UCNP nanoplatforms showed a silicon–oxygen bond at 1,095 cm^−1^, amide bond at 1,553 cm^−1^, and benzene ring bond at 2,950/2,850 cm^−1^, indicating a successful bond between peptide-BHQ and UCNP@mSiO_2_, confirming the successful synthesis of the UCNP nanoplatforms.

### Loading of RBS and evaluation of the NO release property

After successfully preparing the UCNP nanoplatforms, we investigated their drug-loading capacity and ability to stimulate RBS. Under visible light, RBS experienced photoelectronic transition, ligand structural modification, and NO release (Fig. S4). Modest amounts of RBS were mixed with UCNP nanoplatforms, creating UCNP/RBS nanocomplexes when stirred, while NO released from the UCNPs under NIR upconverted to visible light [38]. Compared with the UCNP nanoplatforms, the marked reduction in UCL intensity of the UCNP/RBS nanocomplexes at 500 to 600 nm (Fig. S5A) suggests that the absorbed UV/green light energy from the UCNPs causes NO release through RBS. Moreover, UV-vis absorption peaks of 500 to 600 nm, characteristic of RBS, were evident in the UCNP/RBS nanocomplexes (Fig. S5B), confirming successful RBS loading in the UCNP nanoplatforms. Moreover, the FT-IR spectrum analysis showed NO bands of RBS at 1,745 cm^−1^ (Fig. S5C), and after RBS loading, the UCNP/RBS nanocomplexes also exhibited NO bonds in conjunction with silicon–oxygen bonds. After establishing the standard curve of RBS by UV-vis (Fig. S6A), the samples containing varying concentrations of RBS showed a maximum loading capacity of 78% (Fig. S6B). Furthermore, the stability test results of the solution indicated that the UCNP/RBS nanocomplexes had good stability in deionized water for 1 week (Fig. S7).

**Fig. 3.**
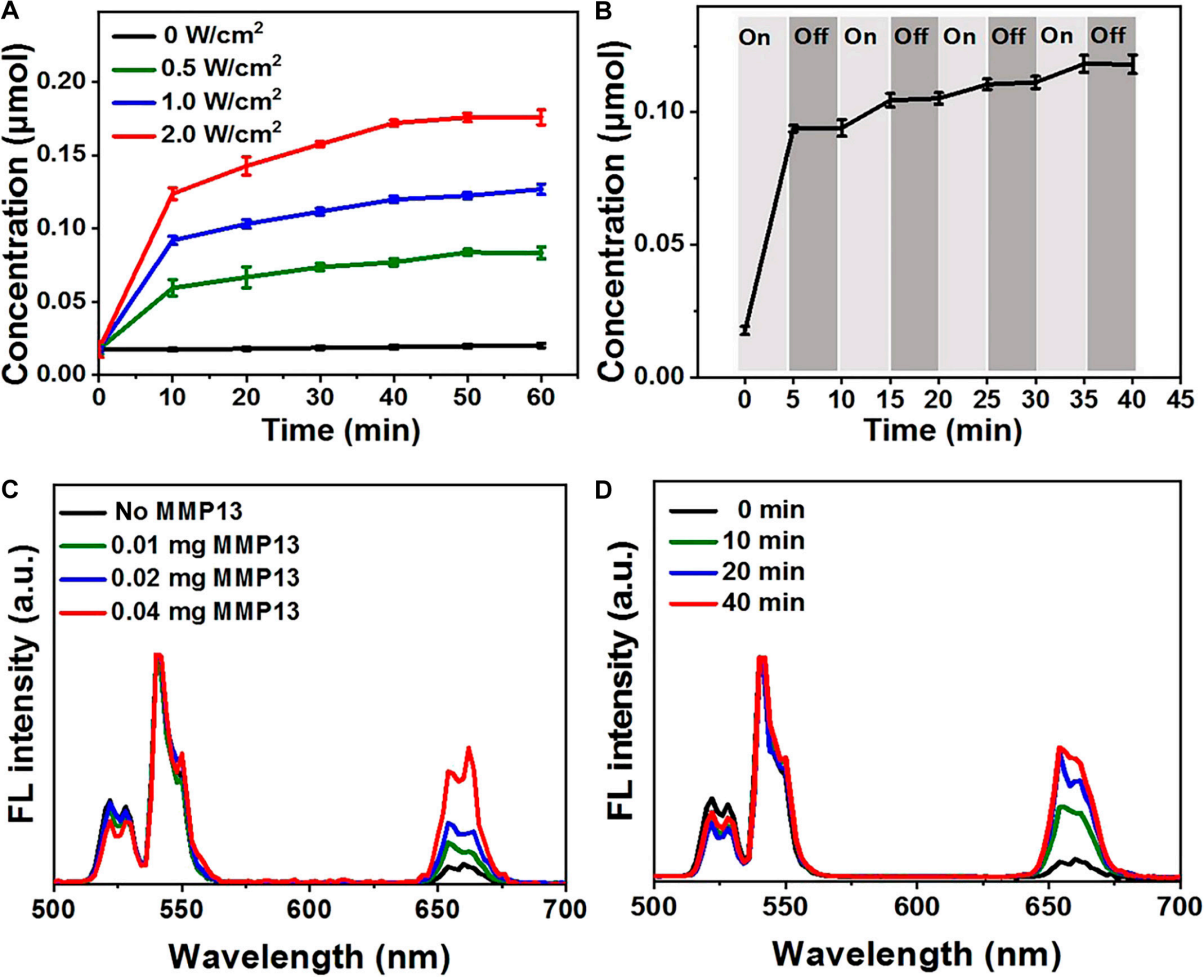
(A) NIR-triggered release of NO from the UCNP/RBS nanocomplexes at different power densities and illumination durations. (B) Turn-on/turn-off behavior of NO release from the UCNP/RBS nanocomplexes via NIR illumination. (C) Fluorescence recovery of the UCNP nanoprobes at 650 nm upon incubation with varying concentrations of MMP13 in an enzyme digestion buffer at 37 °C for 30 min. (D) Fluorescence recovery of the UCNP nanoprobes at 650 nm upon incubation with 0.04 mg of MMP13 for different periods.

We then evaluated the release properties of the UCNP/RBS nanocomplexes under 808-nm NIR irradiation (Fig. 3). Figure 3A shows the excitation of NO by the UCNP nanocomposite platform at 808-nm NIR at 0, 0.5, 1, and 2 W/cm^2^ power, reflecting the release of NO at different power levels. Therefore, no NO release was observed from the UCNP/RBS nanocomplexes at 0 power, but marked NO release was observed when the UCNP/RBS nanocomplex was irradiated with NIR at 0.5 W/cm^2^, reaching a concentration of 0.05 μmol after 10 min of NIR irradiation, and the release of NO continued to increase over the next 60 min and was positively correlated with power density. The concentration of released NO reached 0.1 μmol after 20 min of NIR irradiation when the NIR power was 1 W/cm^2^, which provides a reference for subsequent cell experiments. The concentration of released NO exceeded 0.15 μmol after 30 min of NIR irradiation when the NIR power was 2 W/cm^2^. Next, the on-demand release behavior under repeated switching at 808-nm NIR irradiation was evaluated. The switch of 808-nm NIR laser can control the release of NO. As shown in Fig. 3B, only NIR irradiation triggered NO release, and the release behavior stopped when the NIR power was off, demonstrating that the output power of the 808-nm NIR laser can control the release of NO (on-off and power density). RBS UV typical peak fall further demonstrates that RBS is burned following NIR radiation at 808 nm (Fig. S8). We then measured the temperature of the UCNP aqueous solution under 808- and 980-nm irradiation (Fig. S9). Although a 980-nm laser can excite UCNPs more effectively, the laser-controlled heating effects cannot be ignored. Under 980-nm NIR irradiation, the water temperature rose to 33 °C in only 2.2 min, whereas under 808-nm NIR irradiation, the temperature rose to only 22 °C after 9 min, indicating that the UCNP nanoplatforms excited by 808-nm irradiation can effectively solve overheating problem.

### Detection of MMP13 enzyme activity

With the photoluminescence properties of UCNPs and the conjugation of MMP13-sensitive peptide-BHQ groups, these multifunctional UCNP nanoplatforms can serve as nanoprobes to test the activity of MMP13 by detecting changes in UCNP fluorescence after enzyme digestion [39]. Typically, MMP13 cleaves the peptide sequence Pro-Leu-Gly-Val-Arg-Gly-Lys (PLGVRGK) between Gly and Val [40], resulting in the release of BHQ and the recovery of a 650-nm UCNP fluorescence signal indicative of MMP13 activity. Figure 3C shows that incubating the UCNP nanoprobes with varying concentrations of MMP13 (0, 0.01, 0.02, 0.04 mg) in the enzyme reaction buffer for 30 min at 37 °C leads to a gradual restoration of the 650-nm fluorescence signals depending on the MMP13 concentration under 808-nm NIR irradiation. This indicates the highly sensitive detection of MMP13 by the UCNP nanoprobes, which is facilitated by a highly efficient fluorescence resonance energy transfer (FRET) mechanism between UCNPs and BHQ. The UCNP nanoprobes were also incubated with MMP13 (0.02 mg) for different times (10, 20, 30, and 40 min, at 37 °C; Fig. 3D), and the recovery of the 650-nm fluorescence signal from the UCNPs was directly proportional to the incubation time, demonstrating the efficacy of MMP13 detection by the UCNP nanoprobes. Finally, to validate the specificity of MMP13 detection by the UCNP nanoprobes, the enzyme digestion selectivity of the UCNP nanoprobes was studied by incubating them with different enzymes (BSA, MMP3, MMP7, and MMP13). The results shown in Fig. S10 demonstrate that only incubation with MMP13 led to an appreciable recovery of 650-nm fluorescence from the UCNP nanoprobes as compared with the other enzymes that could only produce a weak recovery. Overall, these findings suggest that the multifunctional UCNP nanoplatforms can be used as effective activatable optical nanoprobes for testing the activity of the MMP13 enzyme and monitoring osteogenic differentiation of MSCs.

### Cell viability and intracellular release of NO

We first texted the cytotoxicity of UCNP/RBS nanocomplexes in MSCs by Alamar blue. As shown in Fig. S11A, the UCNP/RBS nanocomplexes at various concentrations (ranging from 0 to 100 μg/ml) were incubated with MSCs for 24 h and the result indicated the presence of some degree of cytotoxicity with increasing concentrations of the nanocomplexes; however, the survival rate of MSCs exceeded 90% at a concentration of 100 μg/ml across all time points tested. Furthermore, there were no detectable variations in toxicity after a prolonged cell culture time, indicating that the UCNP/RBS nanocomplexes exhibited low cytotoxicity to MSCs. In addition, a long-term toxicity study (24 to 72 h) was conducted to evaluate any potential long-term effects of the UCNP/RBS nanocomplexes, as shown in Fig. S11B. Even after 72 h of incubation with 100 μg/ml UCNP/RBS nanocomplexes, the cell viability of MSCs remained greater than 90%, further substantiating that the UCNP/RBS nanocomplexes exhibited low cytotoxicity to MSCs. Finally, we evaluated the cytotoxicity of MSCs after 808-nm irradiation, as shown in Fig. S12. After incubation with UCNP nanocomplexes, MSCs were explored with 808-nm irradiation of 1 and 2 W/cm^2^ for 5 min and then incubated with live/dead cell detection kit. The cytotoxicity results showed almost no cell death in the NIR irradiation group, indicating that there was no obvious damage to cells by releasing NO from the UCNP nanocomplexes.

**Fig. 4.**
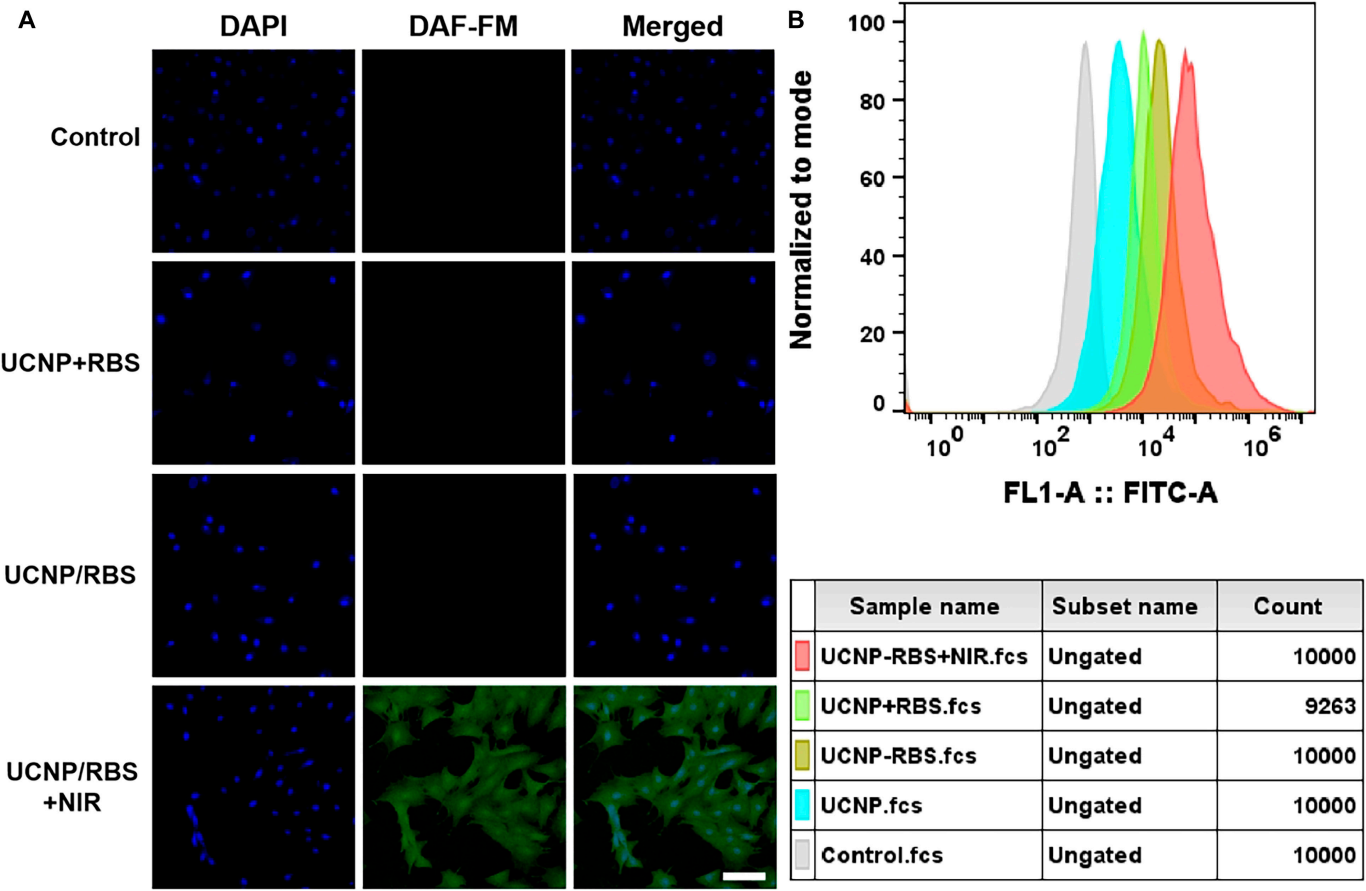
Analysis of NO release properties from the UCNP/RBS nanocomplexes in MSCs. (A) Confocal images of MSCs stained with DAPI (blue) and DAF-FM (NO fluorescent probe, green). These were subjected to various treatments under dark conditions or 808-nm irradiation. Scale bar: 20 μm. (B) Flow cytometric analysis was conducted on DAF-FM intensity in MSCs subjected to different treatments.

Next, the NIR-triggered intracellular release of NO from the UCNP/RBS nanocomplexes was examined. Owing to the short action range of NO, it was imperative to study the intracellular NO release behavior of the UCNP/RBS nanocomplexes within MSCs. A particular NO probe, DAF-FM DA, was used to identify intracellular NO release. DAF-FM DA can easily permeate live cells where it is rapidly catalyzed to transform into DAF-FM, which cannot infiltrate the cell membrane and then reacts with NO to produce intense green fluorescence [41]. As illustrated in Fig. 4, conspicuous green fluorescence was observed only when MSCs were treated with both UCNP/RBS nanocomplexes and 808-nm NIR irradiation (UCNP/RBS + NIR), indicating that the UCNP/RBS nanocomplexes can penetrate MSCs and produce NO in situ under 808-nm laser irradiation (Fig. 4A). Concurrently, flow cytometry results corroborated the findings, further attesting the on-demand NO release property of the UCNP/RBS nanocomplexes within MSCs (Fig. 4B). This ultimately demonstrates NIR-triggered intracellular NO release from UCNP/RBS nanocomplexes under 808-nm laser irradiation.

### NIR-triggered NO release controls osteogenic differentiation of MSCs

**Fig. 5.**
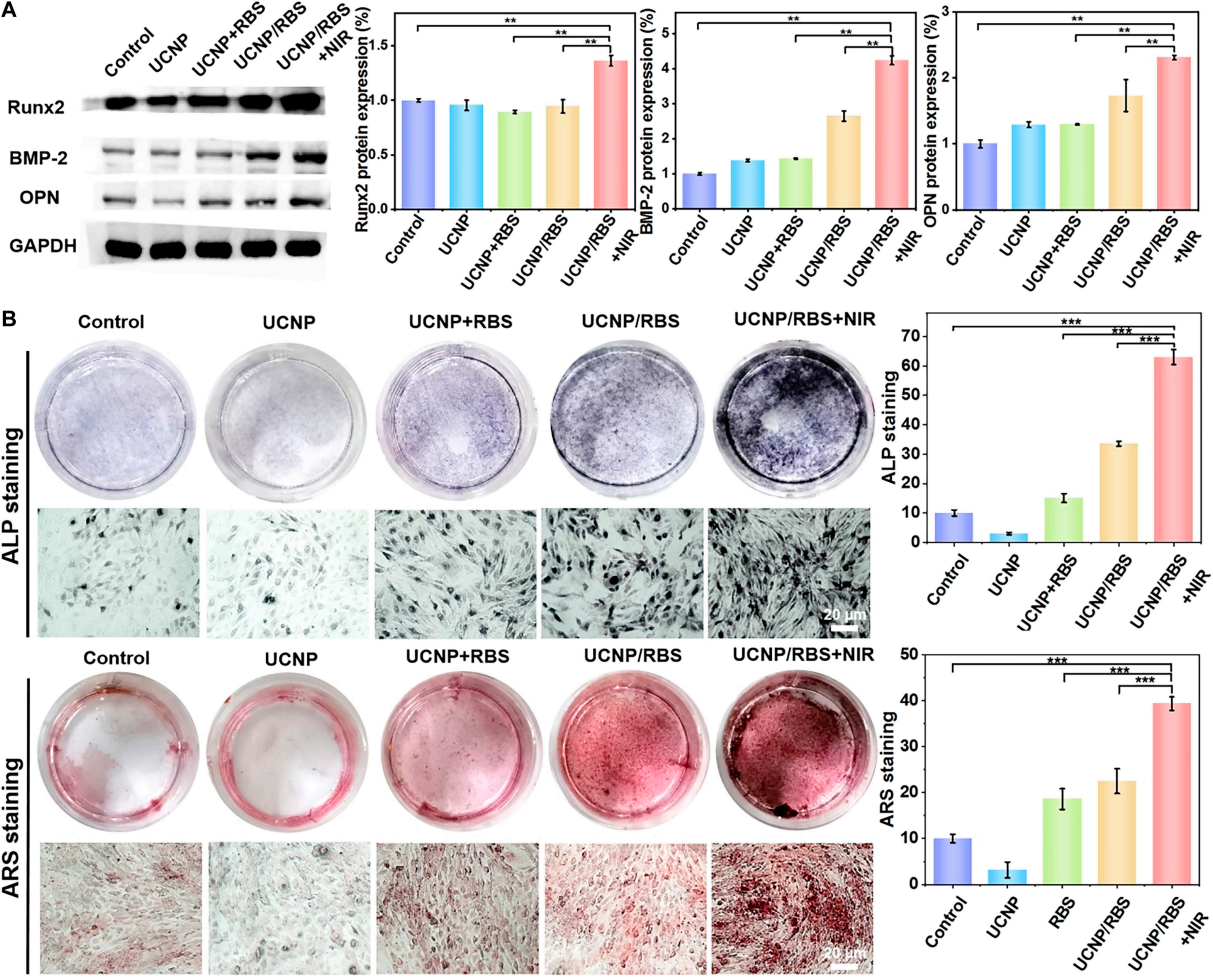
Measurement of osteogenic differentiation in MSCs. (A) Western blot of Runx2, BMP-2, and OPN protein expression in MSCs subjected to different treatments. Statistical analysis of Runx2, BMP-2, and OPN protein expression was performed, and the UCNP/RBS + NIR group showed the highest protein expression. (B) ALP/ARS staining of MSCs with different treatments. The UCNP/RBS + NIR group showed the deepest staining of cells with statistical analysis, demonstrating a marked osteogenic differentiation of MSCs by NIR-triggered release of NO. Statistical significance was evaluated by Student’s *t* test (**P < 0.01, ***P < 0.001). Data are presented as means ± SD.

NO can control and enhance osteogenic differentiation of MSCs but has an extremely short half-life, making it challenging to experimentally achieve an effective concentration in cells. Our UCNP nanoplatforms can load the NO donor RBS to form the UCNP/RBS nanocomplexes and then deliver into MSCs to intracellular triggered release NO by 808-nm NIR light. Through this method, a sufficient level of NO can be obtained within MSCs, which can be used to control and enhance osteogenic differentiation for OP therapy. For the measurement of osteogenic differentiation of MSCs, Western blot was performed first, as illustrated in Fig. 5A. The UCNP/RBS nanocomplexes were incubated with MSCs for NIR irradiation, and then MSCs were cultured to control and enhance osteogenic differentiation by NIR-triggered NO release. Osteogenic differentiation-related expressed proteins such as Runx2, BMP-2, and OPN, which show high expression when MSCs differentiate into osteoblasts [42], were measured by Western blot. The results showed that compared to control group and UCNP/RBS group, the UCNP/RBS + NIR group presented markedly darker and thicker bands for Runx2, BMP-2, and OPN markers. Statistical analysis of Runx2, BMP-2, and OPN protein expression was performed, and the results showed that compared to control, the UCNP/RBS + NIR group showed nearly 1.5 times of Runx2, more than 4 times of BMP-2, and more than 2 times of OPN protein expression. When compared to UCNP/RBS group, the UCNP/RBS + NIR group also showed nearly 1.5 times of Runx2, nearly 2 times of BMP-2, and obviously higher OPN protein expression. These Western blot results confirm that only UCNP/RBS incubation and NIR irradiation could trigger the release of NO to control and enhance the osteogenic differentiation of MSCs.

We further used ALP and ARS staining to evaluate the osteogenic differentiation of MSCs in different groups. ALP is a protein product of osteoblast phenotype and osteoblast differentiation, and the production of calcium nodules also marks osteoblast formation [43]. As shown in Fig. 5B, all cultured samples showed positive ALP/ARS staining, among which the UCNP/RBS + NIR group expressed the highest osteogenesis ability after 10 days of culture (the UCNP/RBS + NIR group showed more than 6 times and 2 times, respectively, when compared to the control and UCNP/RBS group with the statistical analysis by ImageJ), indicating a marked osteogenic effect on MSCs in this treatment group. ARS is used to detect changes in calcium in tissue sections or cultured cells by chelating alizarin red with calcium to form an orange complex [44]. Similarly, ARS staining was also employed to analyze the mineralization stages of osteogenic differentiated MSCs in different groups by staining calcium nodules in red. In accordance with the ARS results, the redness deepened in each group over culturing time based on the gross appearance, and the highest mineralization level with identified distribution of calcium nodules was observed in the UCNP/RBS + NIR group (there was nearly 4 times and 2 times when compared to the control and UCNP/RBS group). It should be noted that because RBS produced trace amounts of NO in the visible light region or under oxygen conditions, the UCNP/RBS group also has osteogenic differentiation of MSCs by the ALP/ARS staining, which was caused by the nonspecific release of NO from UCNP/RBS nanocomplexes [45]. Accordingly, these results indicated that the UCNP/RBS nanocomplexes could remotely control NO release and enhance osteogenic differentiation of MSCs by NIR irradiation.

### Real-time detection of osteogenic differentiation in MSCs

**Fig. 6.**
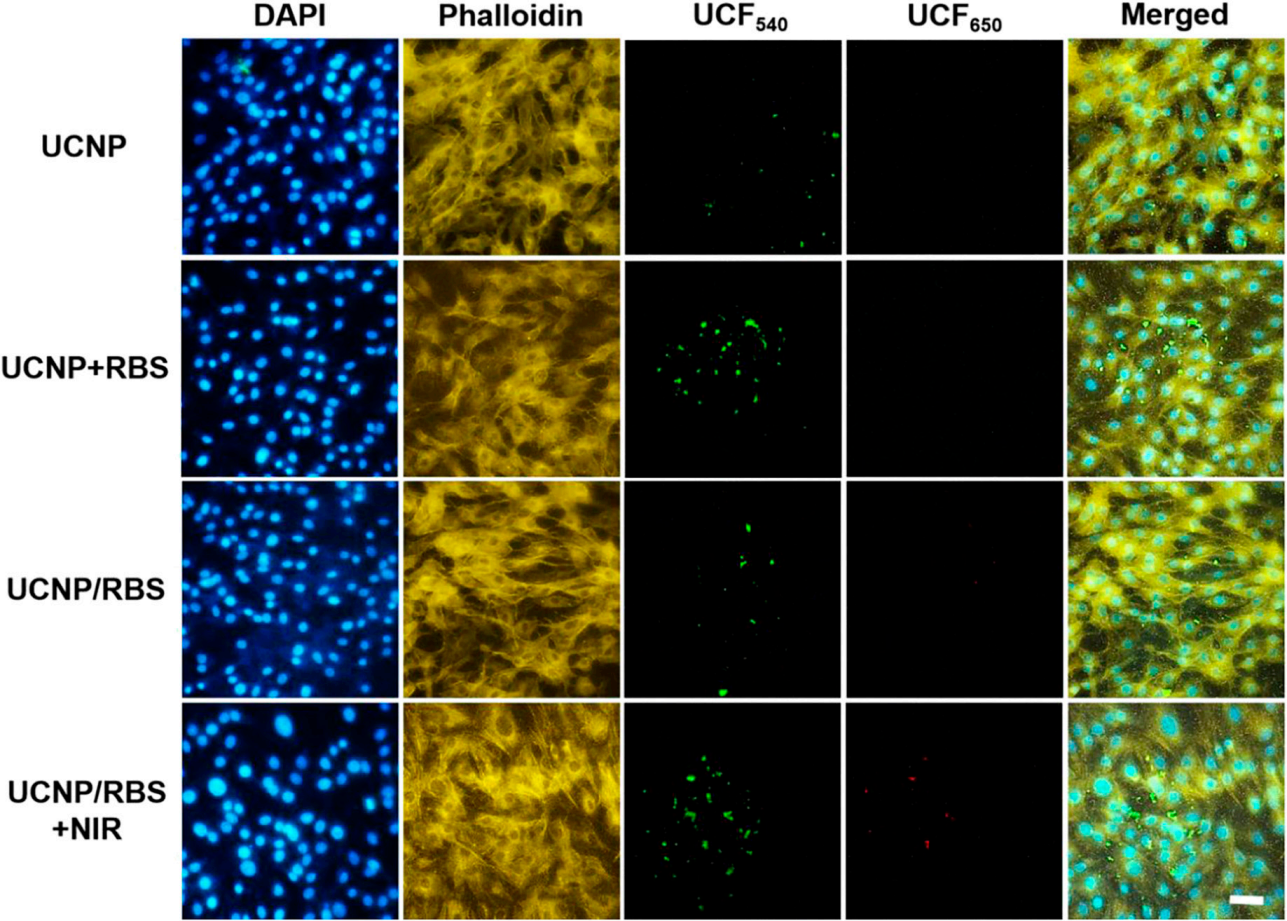
Detection of osteogenic differentiation in MSCs treated with different methods using NIR inverted fluorescence microscopy. Compared with other treatment groups, the UCNP/RBS + NIR treatment group showed marked 650-nm fluorescence recovery under the NIR inverted fluorescence microscope after 7 days of controlled differentiation, demonstrating that the UCNP/RBS nanocomplexes could effectively control and enhance the osteogenic differentiation of MSCs via NIR-triggered release of NO, simultaneously detecting differentiation by enzyme digestion and fluorescence recovery. Scale bar, 20 μm.

The MMP13 enzyme exhibits markedly higher expression when MSCs differentiate in the osteogenic direction, as depicted in Fig. S13. Following the initiation of osteogenic differentiation, the use of UCNP nanoplatforms as nanoprobes allows for the detection of this differentiation through enzyme digestion and fluorescence recovery of UCNPs. As shown in Fig. 6, successful cellular uptake was confirmed, with UCNP nanoprobes seemingly being evenly distributed within the cytoplasm. After subjecting the UCNP/RBS + NIR treatment group to NIR irradiation, a triggered release of NO was observed in the UCNP/RBS nanocomplexes, which in turn controlled the remarkable osteogenic differentiation of MSCs, as demonstrated by the increased expression of MMP13. As a result, more MMP13-sensitive peptides were cleaved from the surface of the UCNP nanoprobes to eliminate the BHQ quencher, thus recovering the 650-nm fluorescence of the UCNP nanoprobes. This enables real-time monitoring of MMP13 activity in differentiated MSCs. However, a mere 1-day induction of MSCs resulted in low enzyme digestion efficiency of the MMP13-sensitive peptide and low fluorescence recovery rate of the UCNP nanoprobes (as shown in Fig. S14), which was due to the low MMP13 expression level in MSCs. After 3 days of induction, the UCNP nanoprobes were able to monitor the osteogenic differentiation of MSCs (indicated in Fig. S14), particularly when NIR was used to mediate NO release from the UCNP/RBS nanocomplexes, thereby controlling and enhancing osteogenic differentiation of MSCs. This finding suggests that the UCNP nanoplatforms can be used as nanoprobes that are capable of fulfilling the requirement for in vitro monitoring of stem cell differentiation and have the potential for in vivo monitoring of osteogenic differentiation to evaluate the therapeutic effect on OP.

### Bone remodeling and OP therapy using UCNP/RBS nanocomplexes

**Fig. 7.**
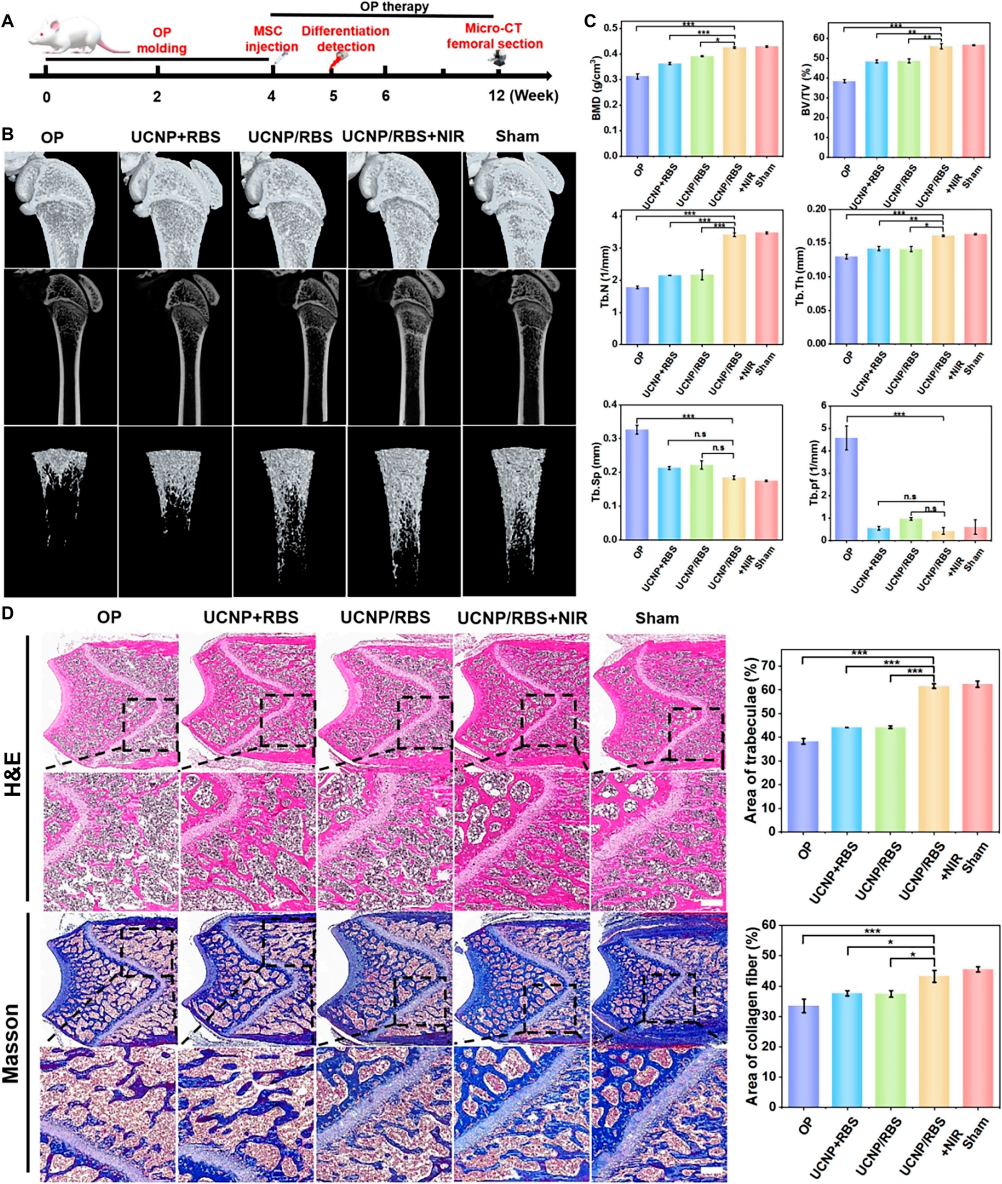
Demonstration of the effectiveness of OP treatment through micro-CT and H&E/Masson’s staining. (A) The timeline of the in vivo experiment is depicted in a schematic illustration. (B) Micro-CT analysis of the femur in OP rats subjected to various treatments after 8 weeks of therapy. Representative images of the OP, UCNP + RBS, UCNP/RBS, UCNP/RBS + NIR, and sham groups are presented from left to right. (C) Architectural parameters, including the bone mineral density (BMD), bone volume fraction (BV/TV), trabecular number (Tb.N), trabecular separation (Tb.Sp), trabecular thickness (Tb.Th), trabecular spacing (Tb.Sp), and trabecular pattern factor (Tb.pf), were measured in different groups after 8 weeks of treatment. (D) H&E staining and Masson’s trichrome staining of rat femoral terminal sections were performed to evaluate the regeneration of bone defects after 8 weeks of treatment with various interventions. Scale bar, 200 μm. Statistical significance was evaluated using Student’s *t* test (*P < 0.05, **P < 0.01, and ***P < 0.001). Data are presented as means ± SD.

A schematic of the animal experiments is presented in Fig. 7A. Initially, osteoporotic models were established by injecting dexamethasone into the right thigh muscle of Sprague-Dawley rats, whereas the control (sham) group received only intramuscular injections of normal saline. This modeling period spanned 4 weeks, after which a micro-CT scan was used to confirm the success of OP induction. As shown in Fig. S15, the group treated with dexamethasone showcased evidence of OP in the femoral head compared with the sham group, indicating the successful generation of the OP model. Subsequently, 20 rats were divided into the following 5 groups for the experimental treatments: the OP group, receiving only MSC injections into the right knee joint cavity; the UCNP + RBS group, where MSCs were incubated with UCNPs and RBS before injection; the UCNP/RBS group, where UCNP and RBS were combined into the UCNP/RBS nanocomplexes, incubated with MSCs, and then injected; the UCNP/RBS + NIR group, where UCNP/RBS nanocomplex-incubated MSCs were injected followed by NIR light irradiation; and the sham group, given only normal saline injections. The treatment process spanned 8 weeks, during which NIR osteogenic differentiation detection was performed after the first week. Previous studies have shown that the efficacy of cell therapy is not hampered by poor engraftment of transplanted cells [46]. A single stem cell therapy session may prove to be ineffective or only modestly effective, but repeated treatments could amplify the benefits. No immunological or infectious complications were recorded throughout the study.

Upon completion of treatment, micro-CT was used to evaluate the bone repair effects over the course [47]. Micro-CT, a 3D imaging technique that employs x-rays to probe small-scale internal structures, also revealed changes in the bone index over time [48]. As shown in Fig. 7B, the femur of the OP group exhibited a porous structure and decreased number of trabeculae compared with those in the sham group. Among the measured bone parameters (BMD, BV/TV, BS/TV, Tb.Sp, and Tb.N), the OP group displayed a substantial decrease in BMD, BV/TV, BS/TV, and Tb.N, coupled with an increase in Tb.Sp, in contrast to the sham group (Fig. 7C). In contrast, the UCNP/RBS + NIR group displayed an improved bone structure, similar to that of the sham group. The UCNP + RBS and UCNP/RBS groups showed marked bone defects and fewer trabeculae. Crucially, the treatment parameters of the UCNP/RBS + NIR group closely mirrored those of the sham group and markedly outperformed the OP, UCNP + RBS, and UCNP/RBS groups. The 808-nm NIR irradiation followed by NO release in the UCNP/RBS + NIR group markedly alleviated the OP condition by controlling and enhancing efficient osteogenic differentiation of MSCs, implying NO as the key promoter of osteogenesis capable of aiding bone mass recovery and reversing OP.

Further investigation of the varied treatment effects on bone structure was conducted using histological staining of regenerative bone sections (Fig. 7D). H&E examination 8 weeks after injection indicated that only a thin layer of soft tissue and relatively small amounts of osteoid were evident in the UCNP + RBS and UCNP/RBS groups. In contrast, the UCNP/RBS + NIR group exhibited marked thickening of trabeculae, increased trabecular bone area, and reduced osteolysis—resulting in considerable osteogenesis. This regenerated bone structure was similar to that in the sham group, thus considerably surmounting the observations made in the OP group. Masson’s staining, which reveals collagen fibers, showed a marked decrease in new bone formation in the OP group. However, after 8 weeks of treatment, we observed some recovery in collagen fiber levels within the femur tissue of the treatment group. Echoing earlier results, the UCNP/RBS + NIR group decidedly outperformed all groups in terms of the most effective promotion of bone tissue regeneration. A marked increase in new bone formation was observed in the UCNP/RBS + NIR group due to the efficient osteogenic differentiation of MSCs controlled by NIR-mediated release of NO, facilitating a more rapid bone regeneration process for OP therapy.

**Fig. 8.**
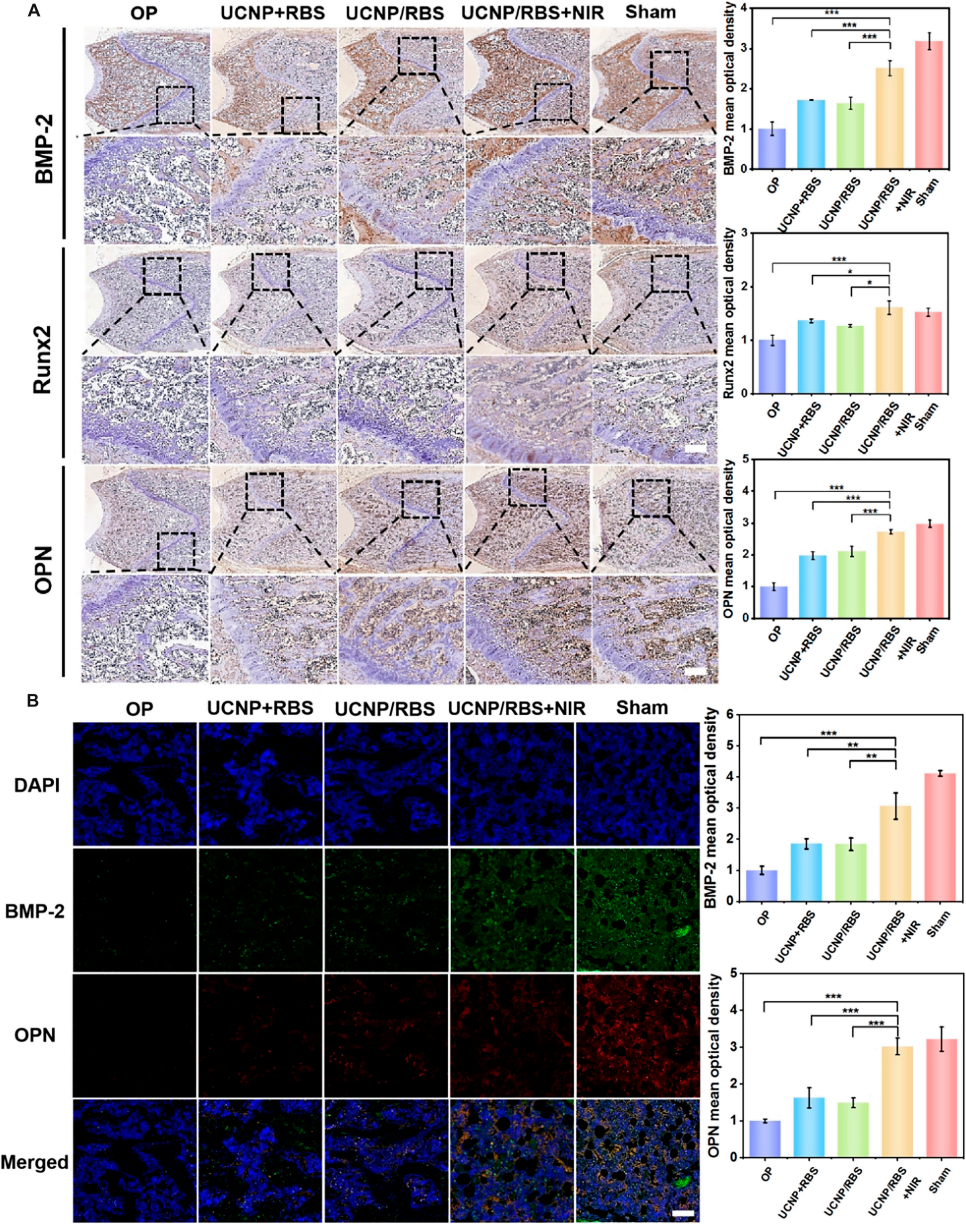
Demonstration of the effectiveness of OP treatment through immunohistochemical/immunofluorescence staining. (A) Immunohistochemical staining of femoral terminal sections with different treatments to demonstrate the expression of osteogenic differentiation-related proteins (BMP-2, Runx2, and OPN) after 8 weeks of treatment. (B) Immunofluorescence staining of femoral terminal sections under different treatment conditions, revealing the expression of osteogenic differentiation-related proteins (BMP-2 and OPN). The fluorescence micrograph of the femur stained for DAPI (blue), BMP-2 (green), OPN (red), and merged. Scale bar, 200 μm. Statistical significance was evaluated using Student’s *t* test (*P < 0.05, **P < 0.01, and ***P < 0.001). Data are presented as means ± SD.

To conclude the efficacy of UCNP/RBS + NIR treatment on accelerating new bone formation and its utility in OP treatment, additional immunohistochemistry analysis was performed after an 8-week treatment period. In this study, we aimed to measure the expression of BMP-2, Runx2, and OPN in the femoral terminal section. Quantitative analysis revealed increased positive expression in the UCNP + RBS, UCNP/RBS, and UCNP/RBS + NIR groups compared with the OP group (Fig. 8A). Protein expression analysis of osteogenic differentiation markers, including BMP-2, Runx2, and OPN, revealed that the UCNP/RBS + NIR group showed markedly augmented marker expression, which was comparable to that in the sham group. Immunofluorescence staining confirmed the differential expression of BMP-2 and OPN proteins in the various treatment groups. Both BMP-2 and OPN markers, which indicate the late stages of osteogenic differentiation in MSCs, were used to further validate the successful differentiation of MSCs into osteoblasts, leading to new bone formation [49]. As depicted in Fig. 8B, confocal images from immunofluorescence staining with BMP-2 (green) and OPN (red) showed muted protein expression in the OP group, indicating minimal osteogenic differentiation and bone remodeling. A similar trend was observed for the UCNP + RBS and UCNP/RBS groups without NO release due to the lack of NIR illumination. However, NIR irradiation triggered UCNP/RBS nanocomplexes to facilitate the release of NO, inducing osteogenic differentiation of MSCs, which eventually led to marked bone repair. This response was especially profound in the sham group, which displayed strong fluorescence of both BMP-2 and OPN proteins, indicating the presence of mature osteoblasts and healthy bone tissue. Overall, these results validate the potency of our developed UCNP/RBS nanocomplexes in efficiently inducing osteogenic differentiation of MSCs in vivo via NIR-triggered release of NO for improved OP.

### Real-time evaluation of osteogenic differentiation in vivo to assess the OP treatment

**Fig. 9.**
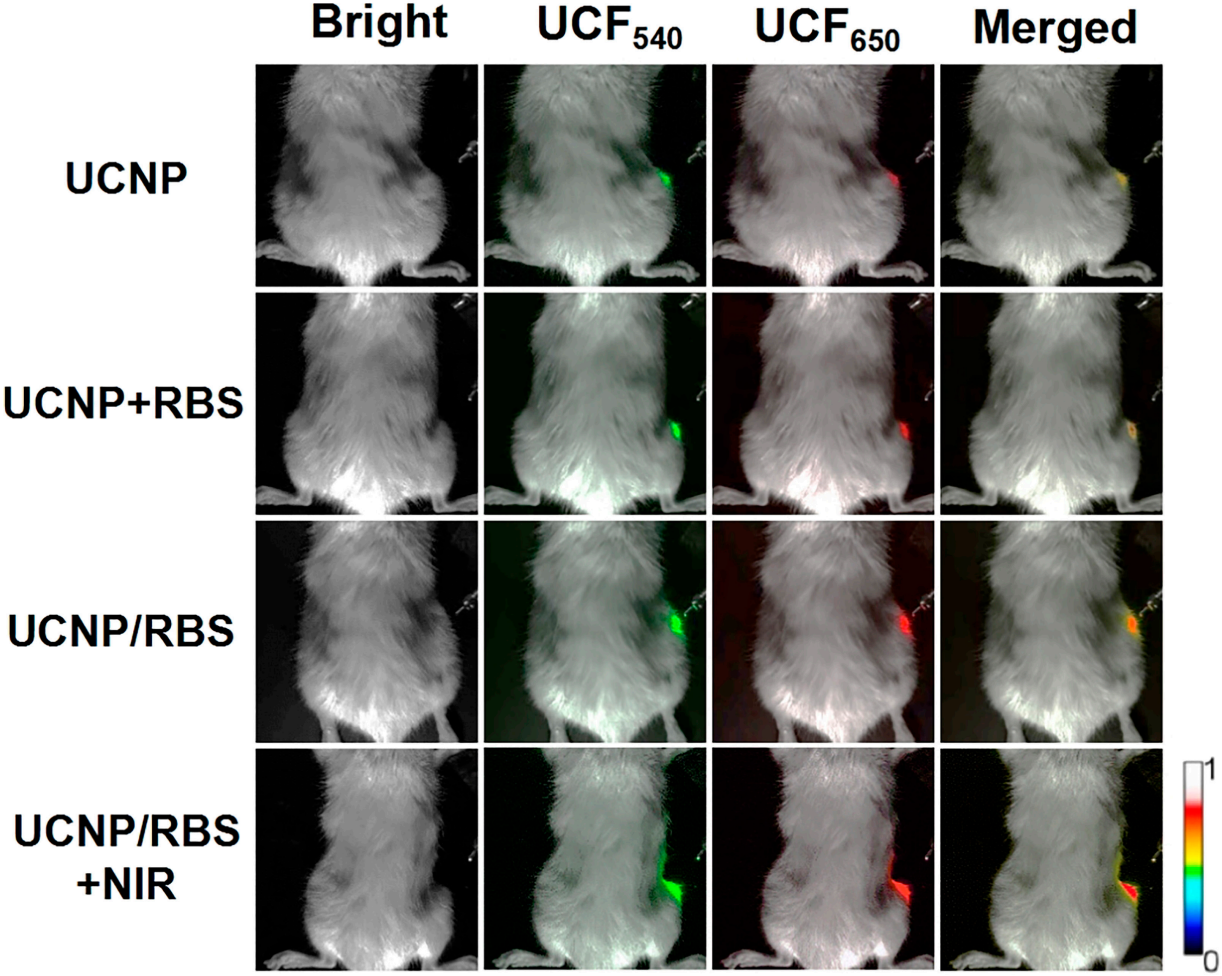
NIR live animal imaging system images of OP rats with different treatments administered by injecting treated MSCs into the right knee after 7 days of feeding. After NIR illumination to control and enhance osteogenic differentiation of MSCs, the UCNP/RBS + NIR treatment group showed strong fluorescence emission of 650-nm recovery, which demonstrated marked osteogenic differentiation of MSCs via NIR-triggered NO release by NIR irradiation. 808 nm: 1 W/cm^2^.

Following the induction of osteogenic differentiation in MSCs, the UCNP nanoplatforms can persist within the cytoplasm, serving as nanoprobes to measure MMP13 activity and monitor MSC osteogenic differentiation in real time in vivo. Based on our in vitro MSC analyses, we evaluated the ability of these UCNP nanoprobes to monitor osteogenic MSC differentiation and offer real-time appraisal of treatment effectiveness in OP rats. OP modeling resulted in OP symptoms at the injection site, which can lead to long-term inflammation, and long-term inflammation can lead to an acidic environment. Interestingly, studies have shown that RBS will further decompose under acidic conditions [50]. Figure 9 illustrates the green (540 nm) and red (650 nm) light signals attributed to MSCs labeled with different groupings during the 7-day culture period. Osteogenic differentiation of MSCs and subsequent bone remodeling triggered by treatment would result in amplified 650-nm fluorescence from the UCNP nanoprobes due to MMP13 enzyme digestion and quencher BHQ removal. Furthermore, UCNPs permitted adequate light penetration, enabling deep tissue imaging and subsequent assessment of the transplanted cell tissue distribution. Fluorescent signals were predominantly concentrated in the area of the bone defect, suggesting that the transplanted MSCs contributed to bone healing. In vivo, a consistent increase in fluorescence intensity was observed following 3 identical cell injections, which was attributable to the responsible accumulation of the UCNP nanoprobes. Significantly, the UCNP/RBS + NIR group demonstrated a marked recovery of 650-nm fluorescence relative to other treatment groups, indicative of efficient MSC osteogenic differentiation prompted by NIR-controlled NO release from the UCNP/RBS nanocomplexes.

## Discussion

Treatment of OP by stem cell transplantation requires the development of a simple and efficient method that can effectively induce and detect osteogenic differentiation of MSCs. OP leads to low bone mass, degeneration of bone tissue, and destruction of bone microstructure. Differentiation of MSCs into bone cells can help in bone regeneration and bone repair, as well as treating OP. At the same time, NO has been shown to have the potential to induce MSCs to differentiate into bone cells. Based on these insights, we have developed an upconversion nanoplatform for effective treatment of OP and detection of osteogenic differentiation of MSCs. There are 2 main ways to overcome the shortcomings of stem cell therapy. First, we developed a vector that can deliver light-sensitive small-molecule RBS to mesenchymal MSCs, photocontrol NO production, and induce MSCs to differentiate into bone cells. Second, it can be used as a probe to detect osteogenic differentiation and evaluate the effect of OP treatment.

NO is known to induce mesenchymal MSCs to differentiate into bone cells. Therefore, we used RBS, a precursor that is simple in synthesis and can be excited by visible light to produce NO, which can be encapsulated on the mesoporous silica layer of UCNP. In addition, in order to effectively detect the therapeutic effect, the MMP13-sensitive peptide and BHQ were connected on UCNP surface to quench the fluorescence, while MMP13 enzyme produced after NO-induced osteogenic differentiation would cut off the sensitive peptide and restore fluorescence to detect the osteogenic differentiation effect.

This study demonstrates the potential of UCNPs in combination with MSCs as an OP drug and for real-time monitoring of osteogenic differentiation of MSCs. Chemical characterization results showed that UCNPs and RBS UCNPs could form stable UCNPs/RBS. NIR light-induced RBS photolysis and NO release experiments showed that UCNPs could effectively release NO under NIR light irradiation, and the release efficiency showed a positive correlation with light intensity and light time. This result laid a foundation for using NIR light to precisely control drug release to induce osteogenic differentiation of MSCs. The detection of MMP13 experiment showed that the fluorescence recovery efficiency of UCNPs fluorescent probe was proportional to the concentration of MMP13 enzyme and incubation time through enzyme digestion reaction and FRET effect, and MMP13 enzyme could be specifically detected. This result provided potential for the use of UCNPs fluorescent probe to detect MMP13 enzyme activity and osteogenic differentiation of MSCs in vivo and in vitro. In vitro cell experiments by cytotoxicity, Western blot, ALP, and ARS cell staining further demonstrated that UCNP-endocytosed cells could effectively release NO and induce osteogenic differentiation of MSCs under NIR irradiation. An OP rat model was established in in vitro animal experiments.

To evaluate the therapeutic effect of different treatment groups, bone tissue was quantitatively analyzed by various micro-CT, H&E, Masson, immunohistochemical, and immunofluorescence staining after 8 weeks of treatment. The results showed that the light group (UCNP/RBS + NIR) showed better therapeutic effect than the non-light group (UCNP/RBS), which verified that the release of NO induced by NIR effectively induced osteogenic differentiation of MSCs to alleviate OP symptoms in OP rats and increased the expression of osteogenic markers of MSCs. At the same time, UCNPs can also be used as fluorescent probes to detect osteogenic differentiation of MSCs in vivo in real time after photocontrolled induction of MSCs. These experimental results indicate that injection of UCNP-ingested MSCs and NIR light excitation can effectively induce osteogenic differentiation and real-time detection.

The UCNP-based NIR photoresponsive NO gas therapy nanoplatform we highlighted in this study to control and enhance osteogenic differentiation of MSCs for OP therapy is as follows: First, osteogenic differentiation of MSCs is induced by NIR photocontrolled NO release. Second, the MMP13-sensitive peptide and BHQ connected on the surface of upconversion nanocomposites can be cut off by MMP13 enzyme produced after osteogenic differentiation of MSCs, and 650-nm recovery can be detected by fluorescence recovery, which has the potential to evaluate the treatment effect of OP.

Although we only studied a local OP treatment model, our upconversion nanoplatform has been shown to be effective in inducing and detecting osteogenic differentiation as a drug carrier and fluorescent probe, and has shown marked therapeutic effect on OP when combined with MSCs. This indicates that it has the potential to be used in OP therapy from local treatment to systemic treatment, and also has promising applications in the field of regenerative medicine.

### Conclusion

In conclusion, we have successfully developed a NIR light-responsive NO gas therapy nanoplatform based on UCNPs to control and enhance osteogenic differentiation of MSCs for OP therapy. The introduction of UCNP nanoplatform can convert NIR light into visible light, which can effectively excite RBS to produce NO while overcoming the limited penetration of UV/blue light in deep tissue, which controls and enhances osteogenic differentiation of MSCs in vitro and in vivo to efficiently treat OP. Moreover, because of the doping of Nd^3+^, the UCNPs can be excited by 808-nm NIR irradiation, effectively reducing the overheating effect caused by 980-nm NIR irradiation. Additionally, on the basis of MMP13-sensitive peptide-BHQ modification and luminescence properties, this UCNP system exhibits a favorable detection property of osteogenic differentiation both in vitro and in vivo for evaluating the treatment effect of OP. Therefore, this multifunctional UCNP nanoplatforms provide a simple and efficient strategy for light-controlled therapy in OP treatment by stem cell therapy.

## Ethical Approval

All animal experiments were approved by the Institutional Animal Care and Use Committee, South China Normal University (ethical number: SCNU-BIP-2023-029).

## Data Availability

The datasets used and/or analyzed during the current study are available from the corresponding author on reasonable request.
