# Supplementary material for: NIR-Triggered Release of Nitric Oxide by Upconversion-Based Nanoplatforms to Enhance Osteogenic Differentiation of Mesenchymal Stem Cells for Osteoporosis Therapy
Source: Biomater Res. 2024 Jul 22;28:0058. doi: 10.34133/bmr.0058 (PMC11260887; doi:10.34133/bmr.0058)
Supplement: Supplementary 1 — Figs. S1 to S15 [file bmr.0058.f1.docx]

**Supporting Information**

**NIR-triggered release of NO by upconversion-based nanoplatforms to enhance osteogenic differentiation of mesenchymal stem cells for OP therapy**

Xulu Ma^1^, Zhao Luan^1^, Qingxin Zhao^1^, Anli Yang^2*^, Jinming Li^1*^

^1^MOE Key Laboratory of Laser Life Science & Institute of Laser Life Science, Guangdong Provincial Key Laboratory of Laser Life Science, Guangzhou Key Laboratory of Spectral Analysis and Functional Probes, College of Biophotonics, South China Normal University, Guangzhou 510631, China.

^2^Department of Breast Oncology, Sun Yat-sen University Cancer Center, State Key Laboratory of Oncology in South China, State Key Laboratory of Oncology in South China, Guangdong Provincial Clinical Research Center for Cancer, Guangzhou 510060, P. R. China.

^*^Corresponding author:

E-mail addresses: yangal@sysucc.org.cn (A. Yang), [lijinm@scnu.edu.cn](mailto:lijinm@scnu.edu.cn) (J. Li)


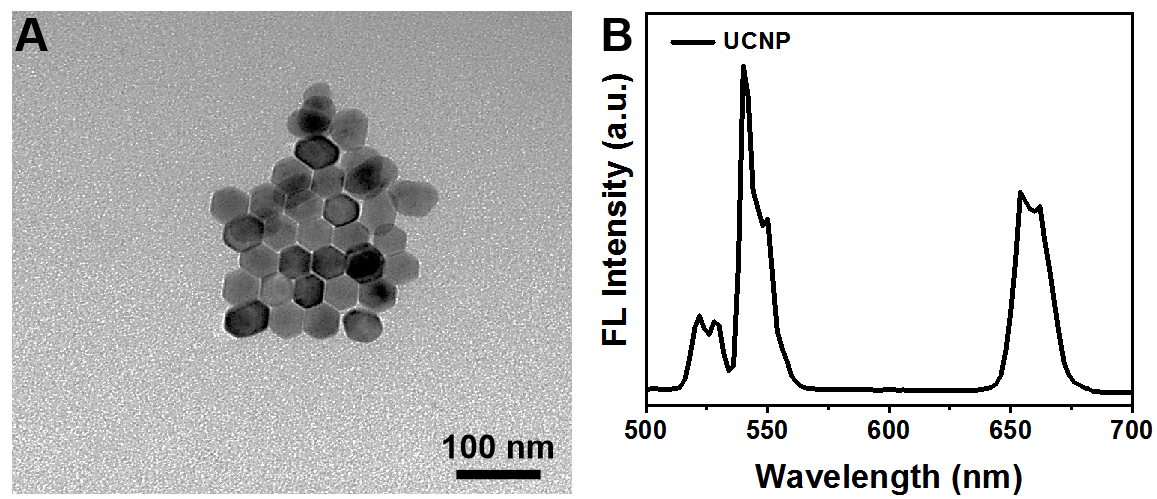


**Figure S1.** Characterization of core-shell UCNPs. A) TEM image of the core-shell UCNPs. B) UCL spectra of UCNPs.

**Figure S2.** Powder X-ray diffraction (XRD) patterns of the UCNP (red line) and the UCNP@mSiO_2_ (gray line) with standard card.


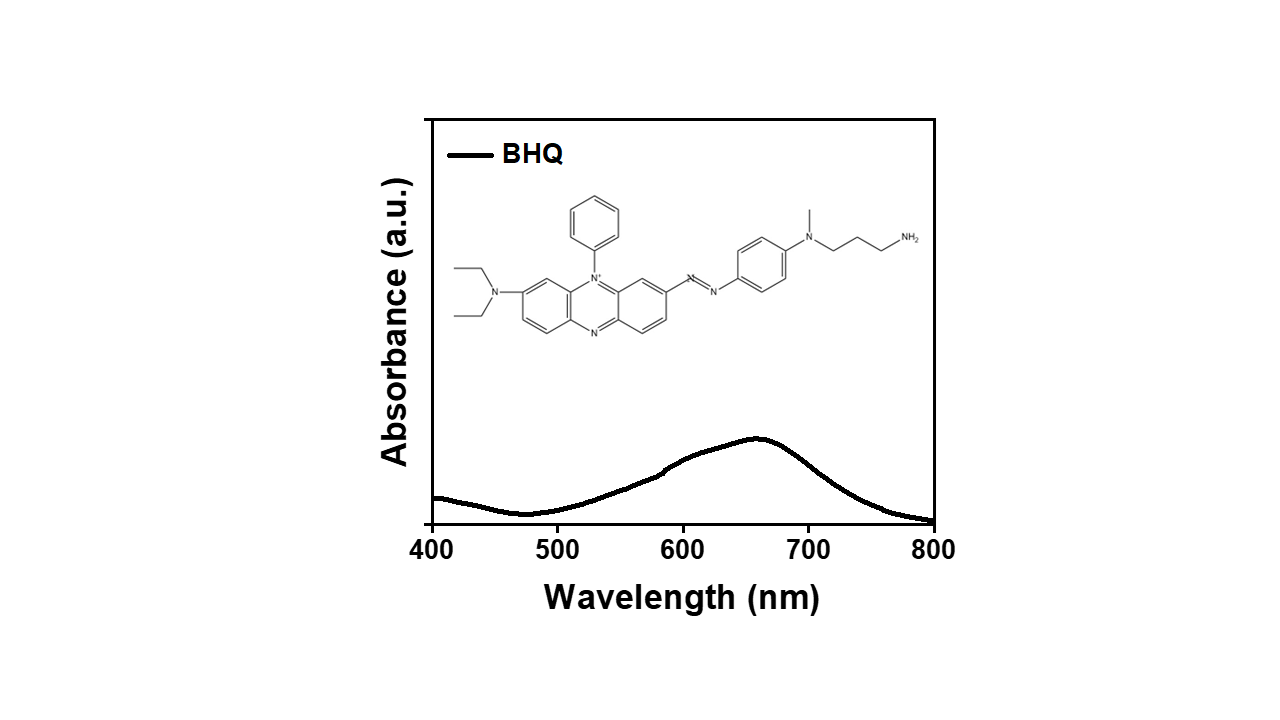


**Figure S3.** The UV absorption and structural formula of BHQ_3_.

**
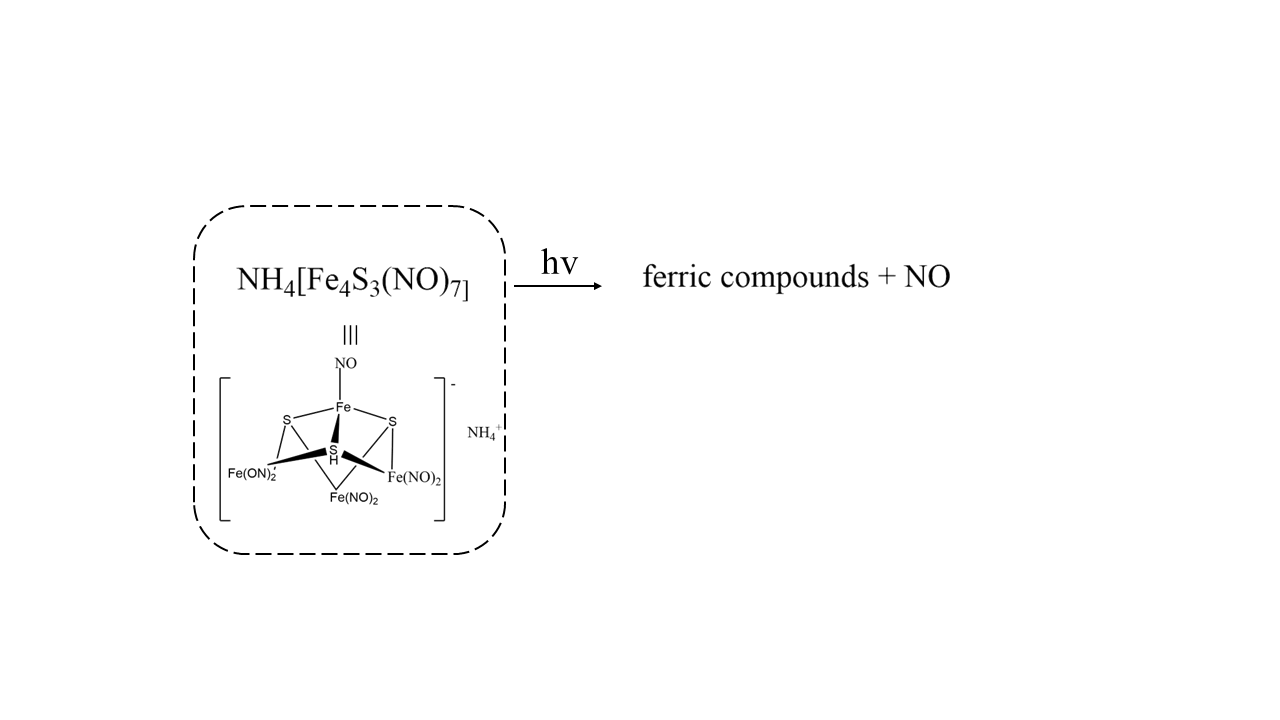
**

**Figure S4.** RBS structure and principle of releasing NO.


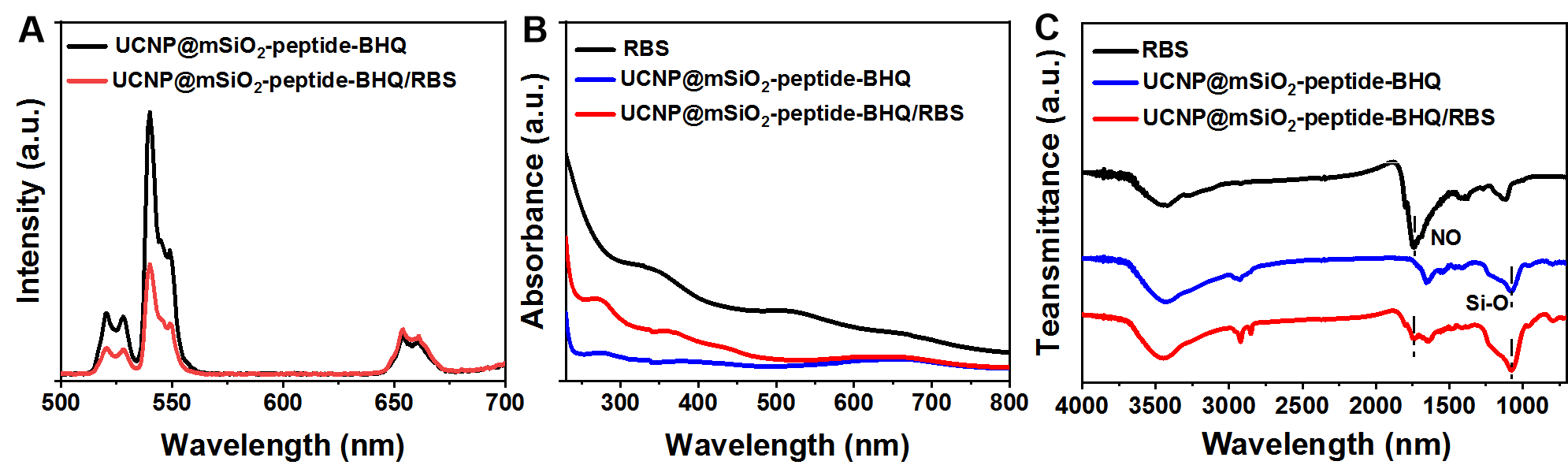


**Figure S5.** The characterization of UCNP/RBS nanocomplexes (UCNP@mSiO_2_-peptide-BHQ/RBS). A) UCL spectra of UCNP@mSiO_2_-peptide-BHQ and UCNP/RBS nanocomplexes. B) UV-vis absorption spectra of RBS, UCNP@mSiO_2_-peptide-BHQ and UCNP/RBS nanocomplexes. C) FT-IR transmission spectra of RBS, UCNP@mSiO_2_-peptide-BHQ and UCNP/RBS nanocomplexes.

**
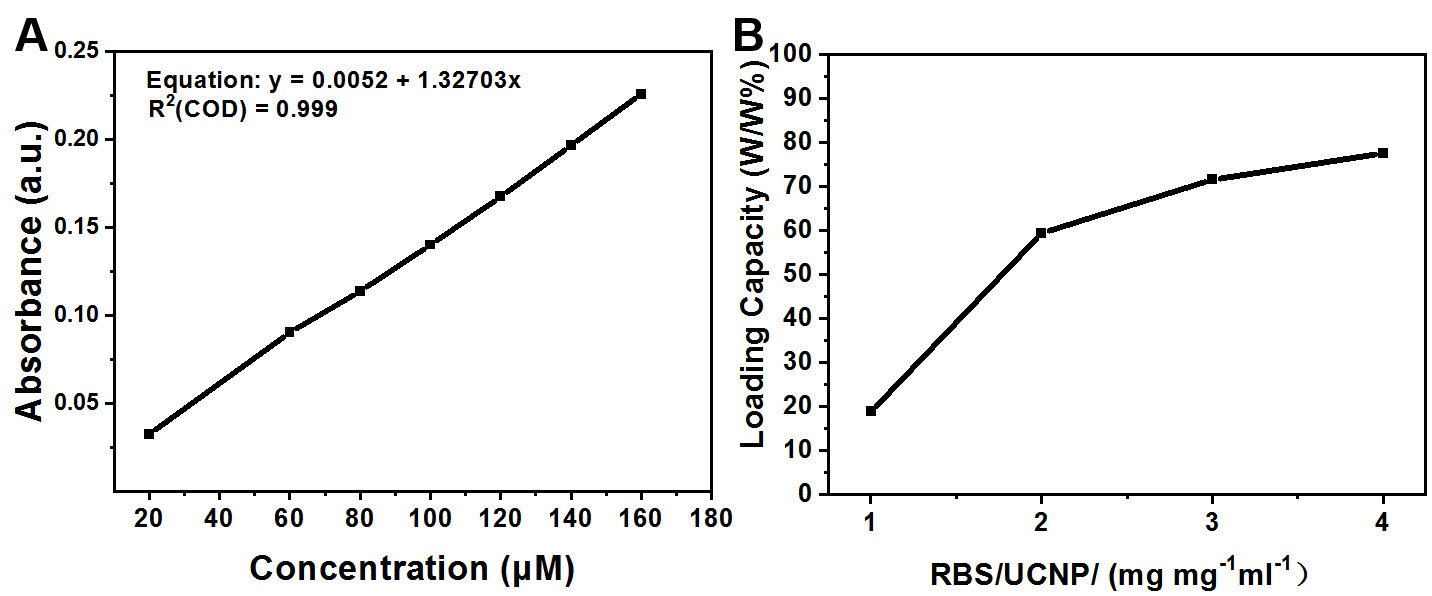
Figure S6.** Standard curve and loading capacity of RBS. A) The standard curve of RBS in ethanol. B) The RBS loading capacity of UCNP nanoplatforms at different weight ratios of UCNP/RBS nanocomplexes.


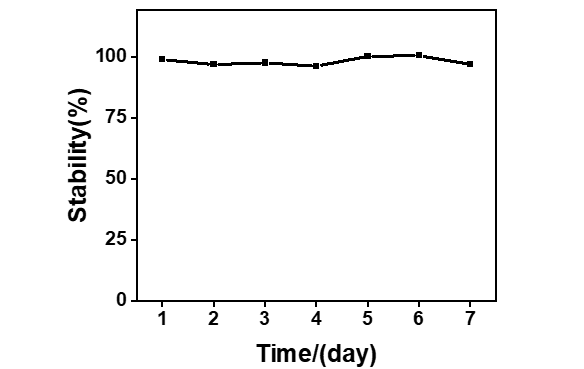


**Figure S7.** The stability of UCNP/RBS in deionized water with long-term colloidal stability.


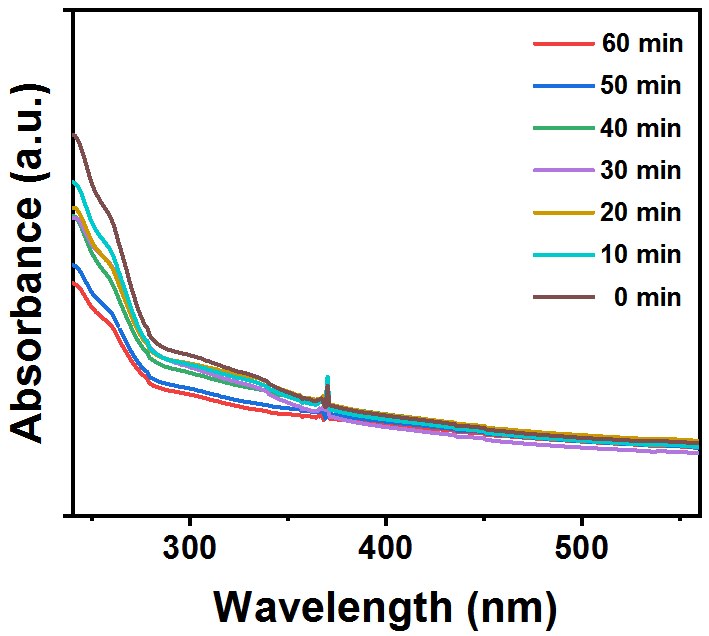


**Figure S8.** The UV absorption spectra of UCNP/RBS nanocomplexes after the 808 nm NIR irradiation (1 W/cm^2^) with different irradiation time (0/10/20/30/40/50/60 min).


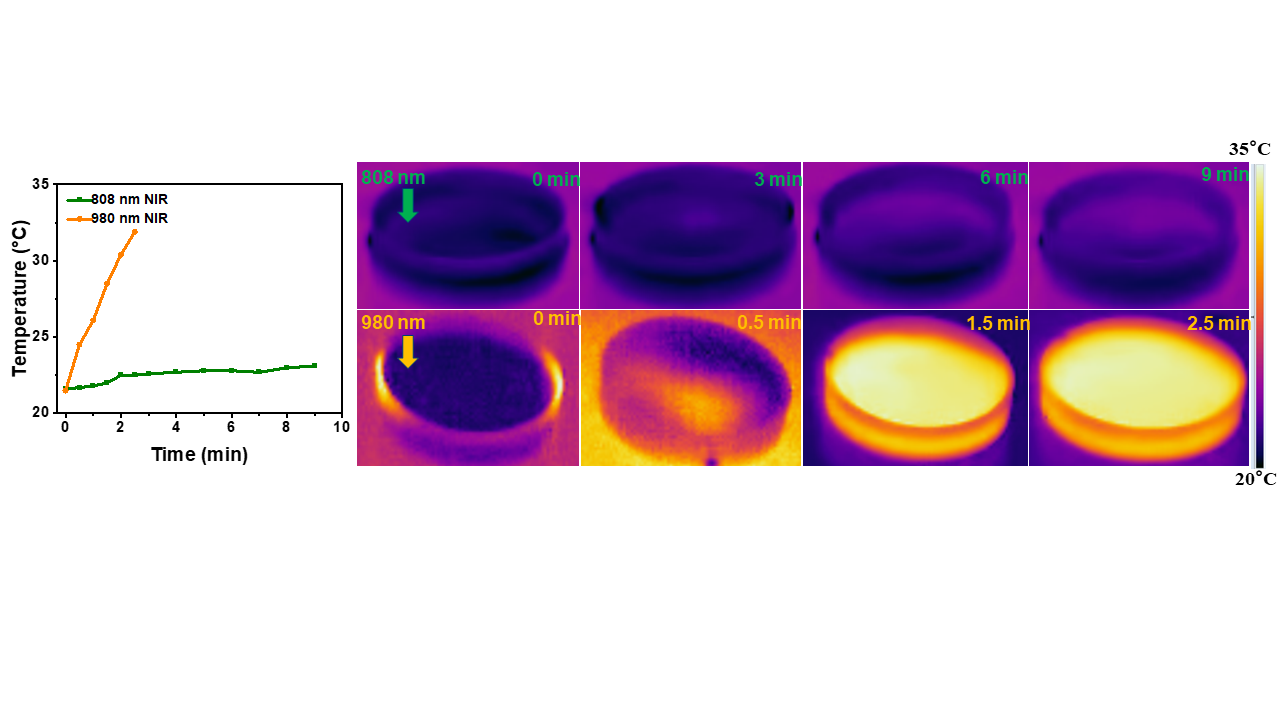


**Figure S9.** The heating curves and thermal imaging real photographs of ultrapure water irradiated by 980 nm and 808 nm laser, respectively.


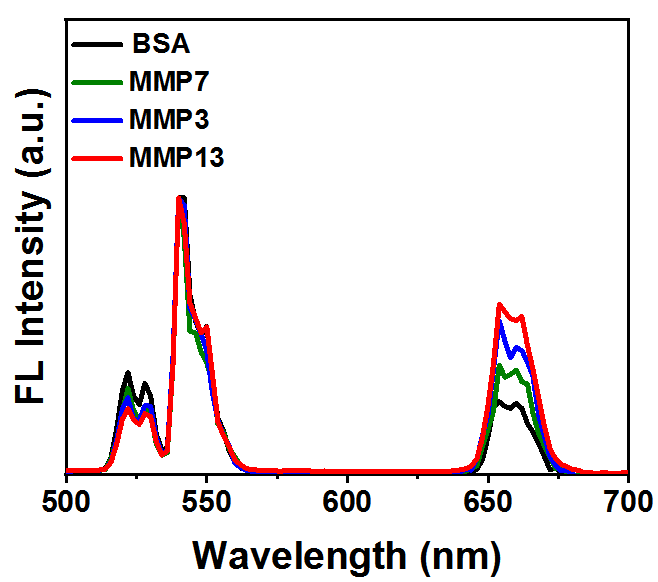


**Figure S10**. Specificity of MMP13 detection by the UCNP nanoprobes. The UCNP nanoprobes were incubated with different enzymes (BSA, MMP 3, MMP 7, and MMP 13), the MMP13 enzyme showed the best fluorescence recovery of 650 nm from UCNPs upon 808 nm NIR light at an intensity of 1 W/cm^2^. n = 3, ^*^*p* < 0.05, ^**^*p* < 0.01.


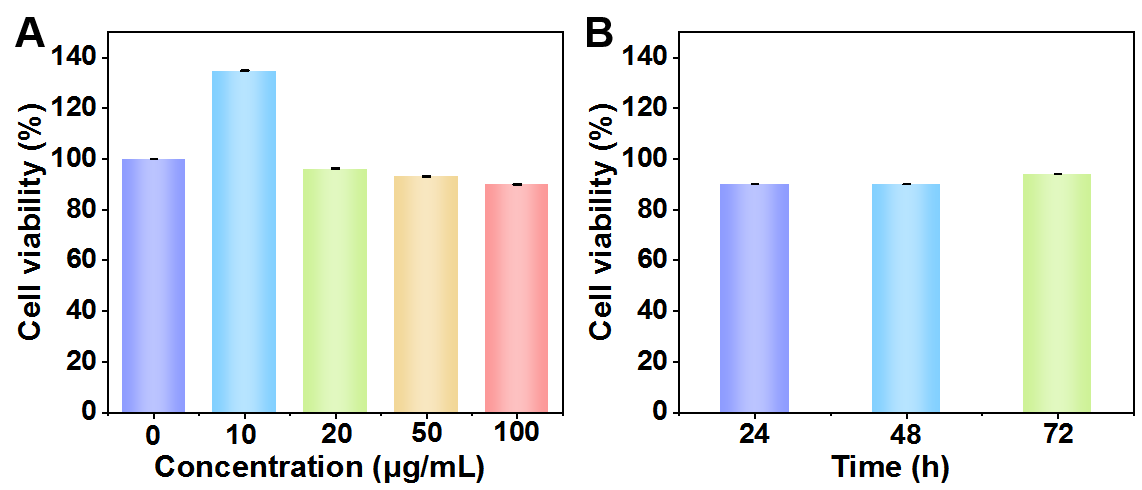


**Figure S11.** Cytotoxicity test of UCNP/RBS nanocomplexes by Alamar blue. A) Cytotoxicity of UCNP/RBS nanocomplexes at different concentrations in MSCs with 24 h incubation. B) Cytotoxicity of UCNP/RBS nanocomplexes at different incubation time (24/48/72 h) in MSCs with 100 μg/mL. Data are means ± s.d. (n = 3)

**
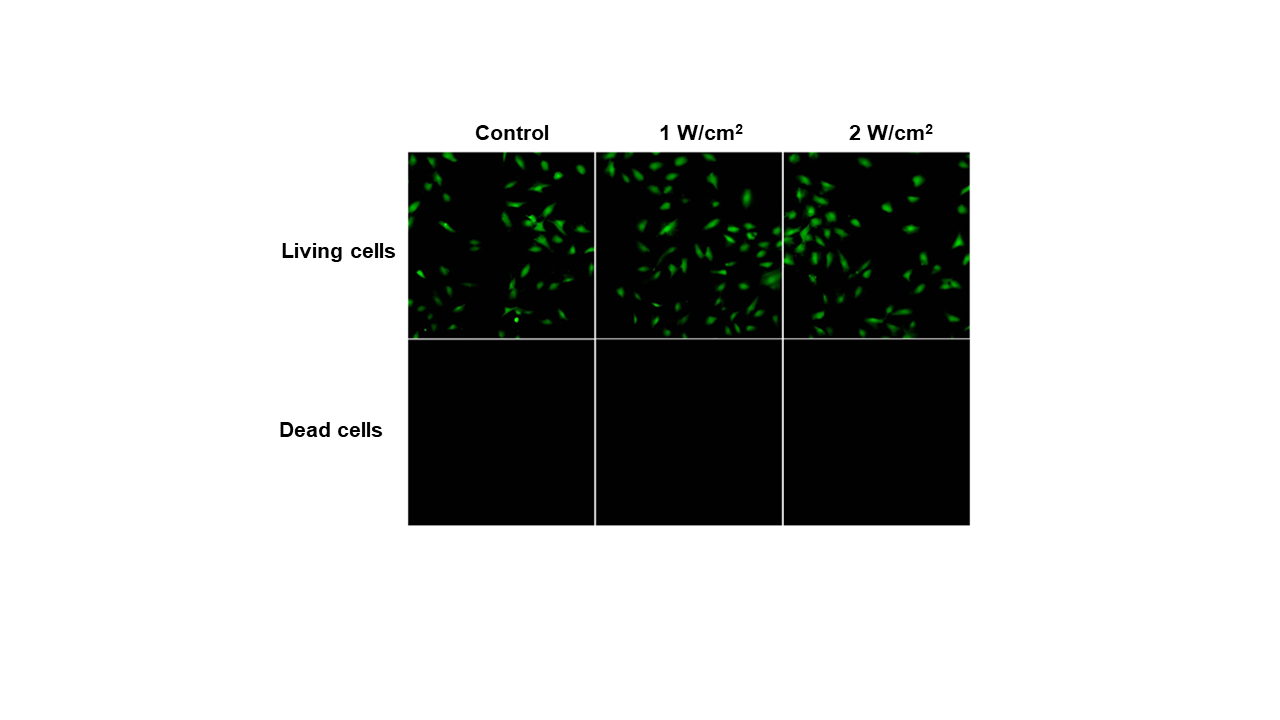
**

**Figure S12.** Cytotoxicity test by live/dead cell kit after NIR irradiation was evaluated.


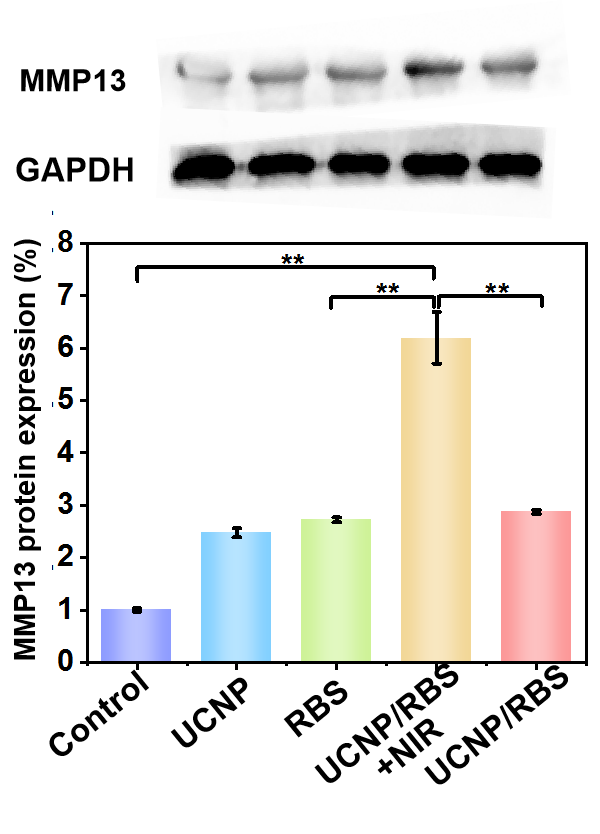


**Figure S13.** Western blot analysis of MMP13 protein expression in MSCs after different treatments and statistical analysis of MMP13 protein expression.

**
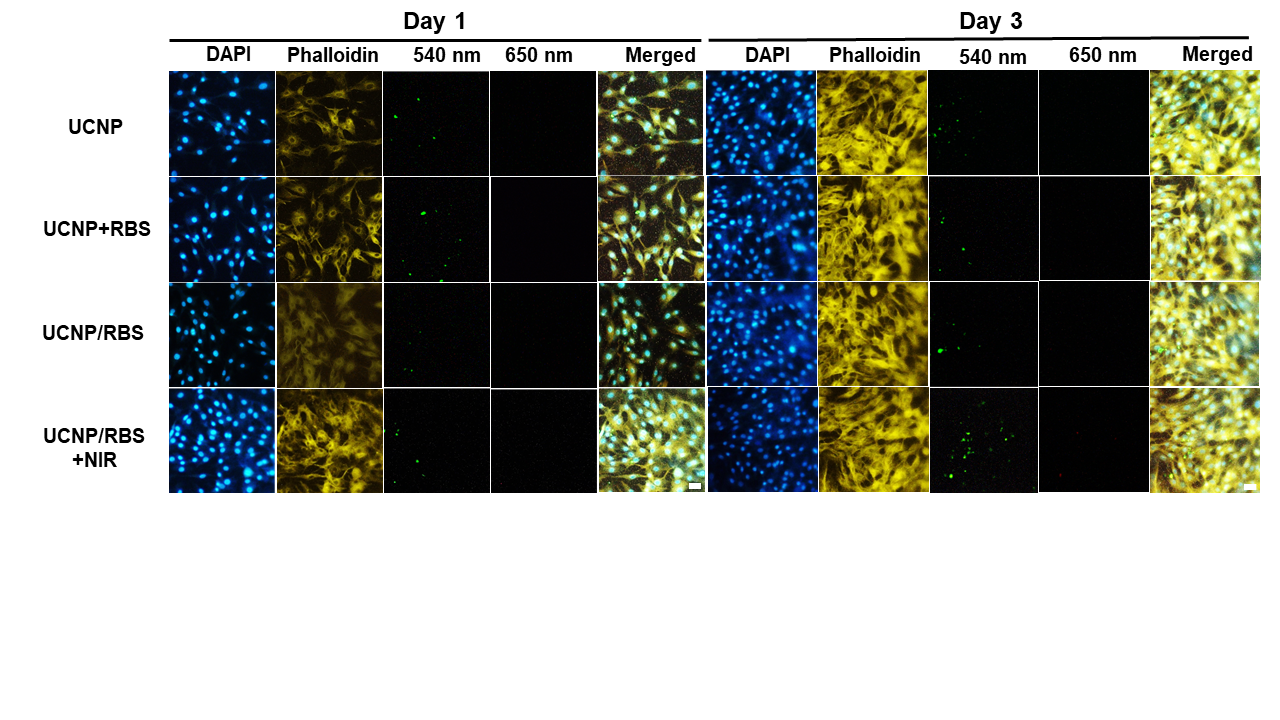
**

**Figure S14.** NIR inverted fluorescence microscope images of MSCs with different treatments for real-time detecting the osteogenic differentiation by UCNP nanoprobes after 1 day and 3 days inducing osteogenic differentiation, respectively. Scale bar: 20 μm.


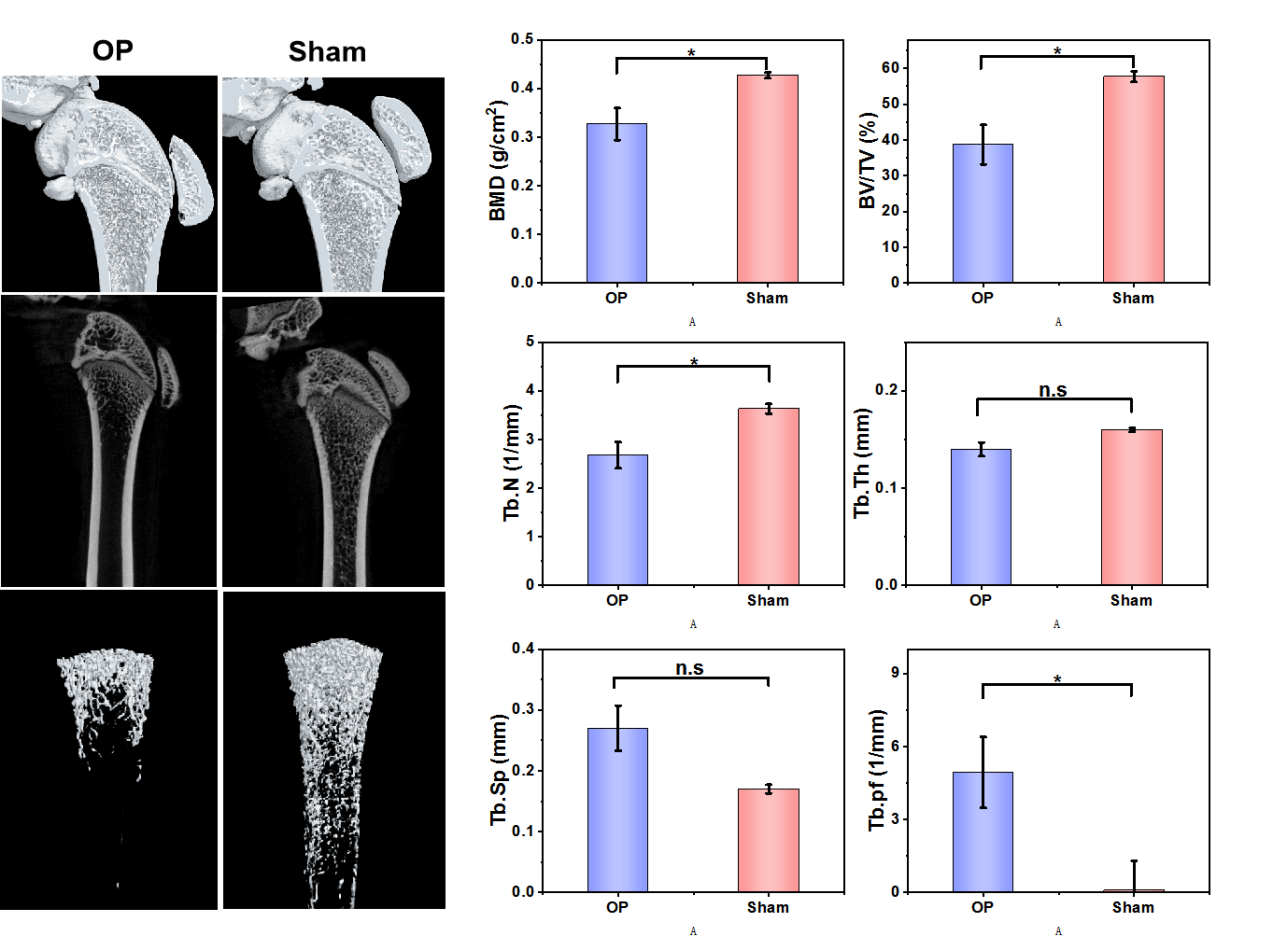


**Figure S15**. Micro-CT images and analysis of OP molding/Sham rat. Dexamethasone (1 mg/kg) was used to inject to the right thigh muscle of SD rat for OP molding. Compared to Sham rat, the femur of OP rat exhibited significant osteoporosis status after four weeks of modeling.
